# Characterising the HLA‐I immunopeptidome of plasma‐derived extracellular vesicles in patients with melanoma

**DOI:** 10.1002/jex2.146

**Published:** 2024-03-26

**Authors:** Caitlin Boyne, Abigail Coote, Silvia Synowsky, Aaron Naden, Sally Shirran, Simon J. Powis

**Affiliations:** ^1^ School of Medicine University of St Andrews Fife Scotland; ^2^ School of Biology University of St Andrews Fife Scotland; ^3^ Biological Sciences Research Complex University of St Andrews Fife Scotland; ^4^ School of Chemistry University of St Andrews Fife Scotland

**Keywords:** cancer immunology, extracellular vesicles, HLA‐I, immunology, immunopeptidomes, tumour associated antigens

## Abstract

Extracellular vesicles (EVs) frequently express human leukocyte antigen class I (HLA‐I) molecules. The immunopeptidomes presented on EV HLA‐I are being mapped to provide key information on both specific cancer‐related peptides, and for larger immunopeptidomic signatures associated with disease. Utilizing HLA‐I immunoisolation and mass spectrometry, we characterised the HLA‐I immunopeptidome of EVs derived from the melanoma cancer cell line, ESTDAB‐026, and the plasma of 12 patients diagnosed with advanced stage melanoma, alongside 11 healthy controls. The EV HLA‐I immunopeptidome derived from melanoma cells features T cell epitopes with known immunogenicity and peptides derived from known tumour associated antigens (TAAs). Both T cell epitopes with known immunogenicity and peptides derived from known TAAs were also identifiable in the melanoma patient samples. Patient stratification into two distinct groups with varying immunological profiles was also observed. The data obtained in this study suggests for the first time that the HLA‐I immunopeptidome of EVs derived from blood may aid in the detection of important diagnostic or prognostic biomarkers and also provide new immunotherapy targets.

## 1 INTRODUCTION

Over the last 30 years, whilst major advances in the treatment options and the understanding of melanoma have developed, the incidence rates of the disease have doubled (Weiss et al., 2019). The first line of treatment for patients with early stage melanoma is surgical resection, however, more than 40% of high‐risk patients that receive surgical excision as the only initial treatment will result in a local or distant disease relapse (Testori et al., 2020). The mutational load of melanoma is known to be high, resulting in melanoma being the most fatal form of skin cancer, despite only being responsible for one percent of all skin cancer diagnoses (Domingues et al., 2018). This high mutational burden leads to altered protein sequences being presented by human leukocyte antigen class I (HLA‐I) proteins and thus promotes T cell responses (Brenner & Hearing, 2008). Melanosomal proteins, such as MART‐1, tyrosinase and gp100 are also frequently recognized by the host T cells, resulting in further activation of a cytotoxic response (Kawakami & Rosenberg, 1996). Melanoma cells have also been demonstrated to regularly re‐express the group of developmental proteins known as cancer/testis antigens, which are also often recognized by the host immune system (Weiss et al., 2019). These various features of melanoma cells enable them to be particularly susceptible to immune modulating therapies. Several such therapies have had clinical success in the treatment of metastatic melanoma, including treatment with immune checkpoint inhibiting monoclonal antibodies targeting CTLA‐4 and PD‐1. The success of these immunotherapies has further highlighted the importance of HLA‐I presentation in effective immune responses against melanoma, and the need for a full characterisation of the immunopeptidome being displayed to the immune system.

Extracellular vesicles (EVs) are small membrane vesicles, typically with a diameter of 50–200 nm that are derived from several sources including the cell surface and from intracellular sources such as multivesicular bodies (MVB). EVs are secreted by almost all human cells into the extracellular space and have the ability to carry bioactive lipids, nucleic acids and proteins as their cargo. The presence of HLA‐I antigen presenting molecules on the surface of EVs, and their ability to present antigenic peptides to T cells, has been well established (Raposo et al., 1996; Théry et al., 1999; Wolfers et al., 2001). Currently, whilst the peptide ligandome (or immunopeptidome) presented by cell surface HLA‐I molecules on cancer cells has been extensively investigated, the EV immunopeptidome remains poorly characterised.

Given that cancer cells are known to release EVs into the circulation, there is reason to believe that such EVs in the blood of patients with cancer could provide information and insight into the state of disease. EVs have already been studied extensively as possible cancer biomarkers to measure disease progression with substantial focus being placed on protein expression. Peinado et al. (2012) found that EVs sourced from the blood of patients diagnosed with stage IV melanoma commonly expressed a number of proteins including oncoprotein MET, very late antigen 4 (VLA‐4), heat shock protein 70 (HSP70) and the melanoma‐specific protein tyrosinase‐related protein‐2 (TYRP2). Further, the expression of scaffolding protein caveolin‐1 on EVs found in the blood, is also considered a biomarker for melanoma as Logozzi et al. (2009) found that melanoma patient plasma EVs were enriched for this protein. Whilst protein expression of cancer patient blood EVs has been well described as a potential source of biomarkers, the HLA‐I immunopeptidome of EVs isolated from the blood of patients with cancer is yet to be extensively defined. In recent work we have shown that the immunopeptidome of EVs derived from breast cancer cell lines does indeed contain known tumour‐associated T‐cell epitopes and immunogenic peptides (Kumar et al., 2022). In this study, we now show that we can detect the immunopeptidome of EVs derived from melanoma patient blood, and that they can also provide information related to the functionality and immunogenicity of the peptides detected on EVs.

## 2 EXPERIMENTAL PROCEDURES

### 2.1 ESTDAB‐026 cell line and biological samples

Melanoma cell line ESTDAB‐026 was obtained from Prof Graham Pawelec and the European Searchable Tumour cell Bank Database (Tubingen, Germany). Twelve melanoma patient samples were obtained from the Manchester Cancer Research Centre Biobank and were characterised as Stage III or Stage IV melanoma (Table 1). Twelve healthy control blood samples were obtained from within the School of Medicine, University of St Andrews under local ethics permission.

**TABLE 1:** Patient characteristics.

Sample	Diagnosis	Stage	Tumour site	Metastatic (Y/N)
MEL‐1	Malignant melanoma	4	Right axilla	Y
MEL‐2	Malignant melanoma	3/4	Right axilla	Y
MEL‐3	Malignant melanoma	4	Left cheek	Y
MEL‐4	Malignant melanoma	4	Left groin	Y
MEL‐5	Malignant melanoma	4	Left radical neck	Y
MEL‐6	Malignant melanoma	4	Upper back	Y
MEL‐7	Malignant melanoma	3B	Right thigh	Y
MEL‐8	Malignant melanoma	4	Right axilla	Y
MEL‐9	Malignant melanoma	3B	Left axilla	Y
MEL‐10	Malignant melanoma	4	Back	Y
MEL‐11	Malignant melanoma	4	Right thigh	Y
MEL‐12	Malignant melanoma	4	Left mediastinal node	Y

Melanoma patient (MEL‐1‐12) characteristics including histological diagnosis, stage of cancer, site of tumour excised at the time of blood sample retrieval and metastatic status.

### 2.2 Characterisation of cells and extracellular vesicles

For nanoparticle tracking analysis (NTA), ESTDAB‐026 cells were cultured for 24 h in serum free RPMI 1640 medium (ThermoFisher Scientific, UK). The conditioned media was then collected and centrifuged at 283 × *g* for 10 mins at 4°C to remove cells and large debris, and then filtered with 0.22 μm Millex‐GP syringe filters (Merck, UK). NTA was performed using a LM‐10 unit (Malvern, UK) equipped with a 638 nm laser. Three videos of 30 s length were recorded for each sample with shutter speeds of 17 or 30 ms. Data analysis was performed using NTA 2.3 software with detection thresholds of three.

For flow cytometry, cells grown in RPMI 1640 supplemented with 5% FBS (ThermoFisher Scientific, UK) were harvested from culture flasks with a 1–2 min incubation with the addition of trypsin (0.025%)‐EDTA (0.01%) solution at 37°C. Cells were then resuspended in culture medium and centrifuged at 283 × *g* for 10 mins at 4°C followed by resuspension in PFN buffer (PBS, 2% FBS, 0.1% Na azide). Cells were stained with primary mouse IgG anti‐HLA‐A, ‐B, and ‐C monoclonal antibody W6/32 (Parham et al., 1979) for 20 min at 4°C, followed by two washes with centrifugation steps as above in PFN. Cells were then stained with the secondary antibody: FITC‐anti‐mouse IgG (Sigma‐Aldrich UK, F2012) at a dilution of 1:100 for 20 min and washed as above. Control cells received second stage FITC anti‐mouse IgG alone. Cells were analysed on a Merck‐Millipore Guava 8HT flow cytometer with a 488 nm laser using Guavasoft 2.7 software.

For immunoblotting, ESTDAB‐026 cells were grown in 175 cm2 flasks for 48 h in serum free medium. After 48 h, the conditioned media was collected, spun and filtered through 0.22 μm filters as above for NTA. Culture supernatants were then spun at 98,380 × *g* for 2 h using a SW32Ti swing‐out rotor in a Beckman L100 ultracentrifuge. The resulting EV pellets were resuspended in lysis buffer (1% NP40, 150 mM NaCl, 10 mM Tris pH 7.4, supplemented with Pierce mini‐protease inhibitor tablets) to create EV lysates. Cell lysates were prepared concomitantly by lysis of the cells remaining in the culture flasks with the same lysis buffer, and spun at 21,130 × *g* for 10 min at 4°C to remove nuclei and insoluble debris. Protein estimations were performed by Bradford assay (ThermoFisher Scientific, UK). About 3 μg of cell and EV lysates and were electrophoresed on 4%–20% gradient SDS‐PAGE gels (ThermoFisher Scientific, UK) and transferred to nitrocellulose (BA85, ThermoFisher Scientific, UK). Membranes were probed with anti‐CD9, CD63 and CD81 antibodies (ThermoFisher Scientific, UK clones Ts9, Ts63 and M38, respectively) at 1:5000 dilution, anti‐HLA‐B and ‐C mouse monoclonal antibody HC10 (Stam et al., 1986) at 1:1000 dilution and rabbit monoclonal anti‐human calnexin (Abcam UK, ab213243) at 1:1000 dilution. Immunoblot signals were revealed with 1:10,000 diluted IR Dye800cw anti‐mouse or anti‐rabbit IgG (LI‐COR, UK) and were then visualized using a LI‐COR Odyssey scanner.

For immunoblotting of melanoma and healthy control blood samples, 1 mL vials of patient plasma were thawed and centrifuged at 21,130 × *g* for 5 min at 4°C to deplete remaining platelets or debris. For size exclusion chromatography (SEC) 10 mL plastic columns (Thermo Fisher Scientific) sealed at the base with fibre glass frits were loaded with Sepharose CL‐4B beads (Sigma‐Aldrich UK), a second frit placed at the top and the column washed with 20 mL of PBS. About 1 mL of the platelet‐depleted plasma was then loaded onto the column. Once the sample had passed through the top frit, approximately 12 mL of PBS was added to the top chamber and 10 × 1 mL fractions were collected. The optical density (280 nm) of each fraction was then assessed by NanoVue Plus Spectrophotometer (Biochrom, Ltd.). Fractions of interest were spun at 124,436 × *g* for 30 min using a SW55Ti swing‐out rotor in a Beckman L100 ultracentrifuge. The resulting EV pellets were resuspended in 30 μL reducing sample buffer. Plasma derived EVs were electrophoresed on 4%–20% gradient SDS‐PAGE gels (ThermoFisher Scientific, UK) and transferred to nitrocellulose filters (BA85, ThermoFisher Scientific, UK). Membranes were probed with anti‐CD9 antibody (ThermoFisher Scientific, UK clone Ts9) at 1:5000 dilution and anti‐HLA‐B and ‐C mouse monoclonal antibody HC10 (Stam et al., 1986) at 1:1000 dilution. Immunoblot signals were revealed with 1:10,000 diluted IR Dye800cw anti‐mouse (LI‐COR, UK) and were then visualized using a LI‐COR Odyssey scanner. Pixel density was measured using Fiji (ImageJ, https://imagej.net/software/fiji/).

For transmission electron microscopy (TEM) imaging, EV isolated by ultracentrifugation from cell line ESTDAB‐026 as per above were resuspended in 0.1 mL PBS. Plasma EV were isolated by SEC in PBS as described in the next section, and were concentrated by ultracentrifugation at 100,000 × *g* for 30 min in an SW55Ti rotor, followed by resuspension in 0.1 mL PBS. About 50 μL of the EV suspensions were fixed for 10 min by mixing with an equal volume of 4% paraformaldehyde. About 20 μL of these samples were then placed on parafilm and TEM grids (glow discharged Formvar film on copper grid, Agar Scientific, UK) were placed inverted onto the sample for 10 min at room temperature. The grids were then lifted and placed (sample side down) onto 20 μL drops of UA‐Zero EM stain (Agar Scientific, UK) for 10 min at room temperature. The grids were then washed by dipping in a 50 mL volume of PBS three times for approximately 5 s each, and then dried on filter paper at room temperature. Transmission electron microscopy (TEM) imaging and measurements were performed on a FEI Titan Themis operated at 200 kV and equipped with a 4k × 4k Ceta CMOS camera.

### 2.3 EV and cell isolation for HLA‐I peptide isolation

The ESTDAB‐026 cell line was cultured in Falcon five‐layer multi‐flasks with a total surface area of 875 cm^2^ (ThermoFisher Scientific, UK) with RPMI 1640 supplemented with EV depleted 2.5% foetal bovine serum (FBS) and 50 μg/mL kanamycin (all ThermoFisher Scientific, UK). The FBS was depleted of EVs by ultracentrifugation at 98,380 × *g* for 4 h at 4°C using a SW32Ti rotor in a Beckman L100 ultracentrifuge, followed by 0.2 μm filtration. When the culture flasks reached 80% cell confluence, fresh culture medium was added to flasks and the cell culture supernatant was harvested every 48–72 h. EV supernatant harvests were 0.2 μm filtered and spun at 98,380 × *g* for 2 h using a Sw32Ti rotor and the EV pellet lysed in lysis buffer (1% NP40, 150 mM NaCl, 10 mM Tris pH 7.4, supplemented with Pierce mini‐protease inhibitor tablets), and stored at −20°C until immunoprecipitation. Cell harvesting was carried out by brief incubation with trypsin‐EDTA solution at 37°C and cells spun at 300 × *g* for 10 min at 4°C. Cell pellets were lysed in lysis buffer as above, spun at 21,130 × *g* for 10 min at 4°C to remove nuclei and debris and the cell lysates were stored at −20°C until immunoprecipitation.

For the melanoma and healthy control blood samples, as above for immunoblotting, 1 mL vials of patient plasma were thawed and centrifuged at 21,130 × *g* for 5 min at 4°C to deplete platelets or debris. SEC 10 mL plastic columns were prepared with Sepharose 4B as above. Samples of plasma were then loaded into the column. 10 × 1 mL fractions were collected as above. Optical density of each fraction was then assessed by NanoVue Plus Spectrophotometer (Biochrom, Ltd). Fraction numbers 4–6 for each myeloma patient sample and 4–7 for each melanoma and breast cancer patient sample were pooled and lysed in lysis buffer (1% NP40, 150 mM NaCl, 10 mM Tris pH 7.4) then stored until immunoprecipitation.

### 2.4 Immunoprecipitation and mass spectrometry analysis

The immunoprecipitation protocol was conducted as follows. EV and cell lysates of volumes between 5 and 10 mL in 15 mL tubes were incubated with 0.3–0.5 mL W6/32‐coupled Protein G‐Sepharose beads (crosslinked using BS3 according to manufacturer instructions, ThermoFisher Scientific, UK) for 1–2 h. The beads were then pelleted by centrifugation at 283 × *g* for 2 min. This was followed by washing the beads by adding 15 mL wash buffer (150 mM NaCl, 10 mM Tris pH 7.4), inverting the tubes several times to fully resuspend the beads and recentrifugation at 283 × *g* for 2 min. After at least five wash steps the beads were resuspended in 1 mL of 1% trifluoroacetic acid (TFA) for 10 min at room temperature to release HLA‐I and bound peptides. Peptides were then isolated on Pierce C18 100 μL tips (ThermoFisher Scientific, UK, 87784) based on manufacturer instructions. Briefly, tips were wetted twice in 0.1 mL 50% acetonitrile (ACN), and equilibrated twice in the same volume of 0.1% TFA. Samples in 1.0% TFA were then passed through the tip twice. The tip was washed twice in 0.1 mL of 0.1% TFA and then eluted with 0.1 mL of 30% or 40% ACN and 0.1% TFA. For ESTDAB‐026, the peptide fractions were eluted in 30% ACN and 0.1% TFA and dried down by speedvac for mass spectrometry. For the melanoma patient and healthy control biological samples, the peptide fractions were eluted in 40% ACN and 0.1% TFA and dried down by speedvac for mass spectrometry.

Peptide samples were processed by mass spectrometry. ESTDAB‐026 samples peptides were analysed on an AB Sciex TripleTOF 5600+system mass spectrometer (Sciex, Framingham, MA) coupled to an Eksigent nanoLC AS‐2/2Dplus system. The samples were loaded in MS loading buffer (2% ACN and 0.05% trifluoroacetic acid) and bound to an Aclaim pepmap 100 μm × 2‐cm trap (Thermo Fisher Scientific), and washed for 10 mins to waste after which the trap was turned in‐line with the analytical column (Aclaim pepmap RSLC 75 μm × 15 cm). The analytical solvent system consisted of buffer A (2% ACN and 0.1% formic acid in water) and buffer B (2% water with 0.1% formic acid in acetonitrile) at a flow rate of 300 Nl/min with the following gradient: linear 1%−20% of buffer B over 90 mins, linear 20%–40% of buffer B for 30 min, linear 40%−99% of buffer B for 10 min, isocratic 99% of buffer B for 5 min, linear 99 −1% of buffer B for 2.5 min, and isocratic 1% solvent buffer B for 12.5 min. The mass spectrometer was operated in the DDA top 20 positive ion mode on charged ions with 2+ to 5+, with 120 and 80 ms acquisition time for the MS1 (*m*/*z* 400−1250) and MS2 (*m*/*z* 95−1800) scans, respectively, and 15‐s dynamic exclusion. Rolling collision energy was used for fragmentation.

Melanoma and healthy control biological sample peptides were subjected to liquid chromatography with tandem mass spectrometry (LC–MS/MS) using an Ultimate 3000 RSLC (Thermo Fisher Scientific) coupled to an Orbitrap Fusion Lumos mass spectrometer (Thermo Fisher Scientific) equipped with a FAIMS interface. Peptides were injected onto a reverse‐ phase trap (Pepmap100 C18 5 μm 0.3 × 5 mm) for pre‐concentration and desalted with MS loading buffer, at 5 μL/min for 10 min. The peptide trap was then switched into line with the analytical column (Easy‐spray Pepmap RSLC C18 2 μm, 15 cm × 75 μm ID). Peptides were eluted from the column using a linear solvent gradient using the following gradient: linear 4%−40% of buffer MS‐B over 45 min, linear 40%−95% of buffer MS‐B for 4 min, isocratic 95% of buffer MS‐B for 6 min, sharp decrease to 2% buffer MS‐B within 0.1 min and isocratic 2% buffer MS‐B for 10 min. The mass spectrometer was equipped with FAIMS at two voltages (−45 V, −65 V) and operated in DDA positive ion mode with a cycle time of 1.5 s. The Orbitrap was selected as the MS1 detector at a resolution of 60000 with a scan range of from *m*/*z* 375–1500. Peptides with charge states 2–5 were selected for fragmentation in the Orbitrap (resolution: 30,000) using CID as collision energy.

### 2.5 Peptide identification

Peptide identification was achieved with Mascot Server Software (Matrix Science). For peptide identification, the raw data files were converted into MZxml using MSconvert (ProteoWizard) and searched using Mascot with against the Swissprot database, restricted only to proteins from humans (updated monthly). The mass accuracy for the MS scan was set to 20 ppm and for the fragment ion mass was set to 0.1 Da. No enzyme was selected with 4 missed cleavages allowed. Oxidised methionine was selected as variable modification. Both assigned and unassigned peptides were selected for further filtering and analysis.

### 2.6 HLA‐typing

ESTDAB‐026 is HLA‐I typed as HLA‐A02:01, HLA‐A68:01, HLA‐B15:01, HLA‐B44:02, HLA‐C03:03, HLA‐C07:04, as determined by the Immune polymorphism database (https://www.ebi.ac.uk/ipd/estdab/). In the absence of HLA‐I typing of the melanoma and healthy control blood samples the 19 most common UK HLA‐I alleles were used in subsequent searches (http://www.allelefrequencies.net/pop6001a_gsb.asp). The HLA‐alleles assessed were as follows: HLA‐A01:01, HLA‐A02:01, HLA‐A03:01, HLA‐ A11:01, HLA‐A24:02, HLA‐B07:02, HLA‐B08:01, HLA‐B15:01, HLA‐B18:01, HLA‐B27:01, HLA‐ B35:01, HLA‐B44:02, HLA‐B51:01, HLA‐B57:01, HLA‐C03:01, HLA‐C04:01, HLA‐C05:01, HLA‐ C06:02 and HLA‐C07:02.

### 2.7 HLA‐I peptide binding affinity and data analysis

Eluted peptides identified from each replicate were combined, duplicate peptides were removed and filtered to select 8–15 amino acid long peptides. The total number of 8–15 mer peptides was defined as HLA‐I immunopeptidome of cell and EVs from ESTDAB‐026 and EVs from the blood samples. Peptides present in the HLA‐I immunopeptidome were assessed for their predicted allele‐binding and affinity using algorithm netMHCpan 4.0 (http://www.cbs.dtu.dk/services/NetMHCpan/) (Jurtz et al., 2017) using default settings. Peptides with IC50 up to 1000 nM were considered to have high predicted binding affinity to HLA‐I and were tabulated. Plots of predicted binding affinity of HLA‐I immunopeptidome for ESTDAB‐026 were made using Prism 8 (GraphPad Inc.) software. For identification of immunogenic peptides reported in previous studies and the peptides derived from tumour‐associated proteins, peptides with 8–15 amino acid length were searched against the Tantigen database (http://projects.met‐hilab.org/tadb/index.php). For analysis of pathway representation within peptide datasets, peptides were matched to proteins using the peptide2protein conversion tool on the piNET server (piNET, LINCS—http://www.pinet‐server.org/pinet/peptideToProtein). Protein lists were then compiled by cancer type and a statistical overrepresentation test was carried out using PANTHER classification system (PANTHERdb V17.0, http://www.pantherdb.org/about.jsp). The annotation data set Reactome Pathways was employed and Fisher's Exact Test with Bonferroni correction for multiple testing were applied to assess significance.

## 3 RESULTS

### 3.1 Characterisation of ESTDAB‐026 melanoma cell line and extracellular vesicles

**FIGURE 1:**
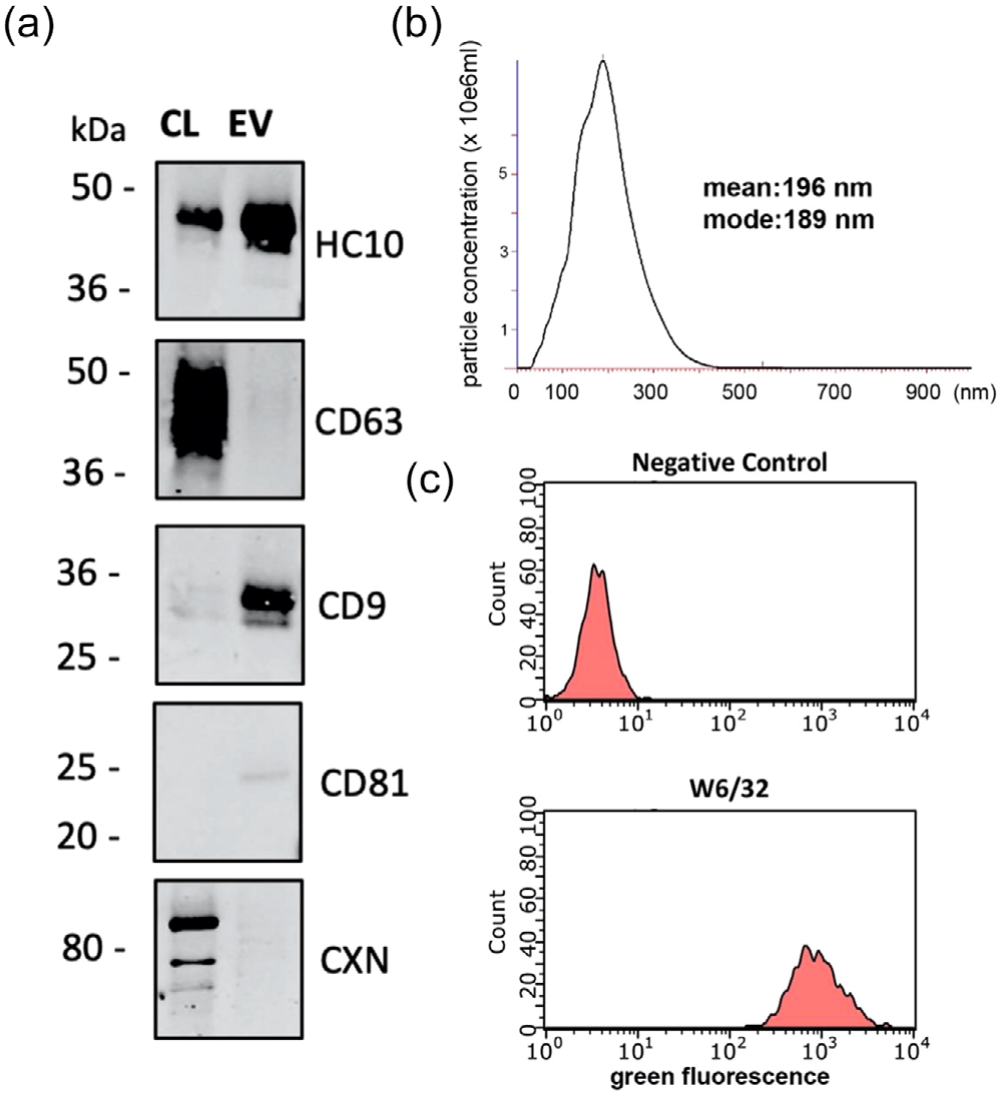
Characterisation of extracellular vesicles derived from the melanoma cancer cell line: ESTDAB‐026. (a) Immunoblotting analysis of detergent cell lysates (CL) and EV lysates (EV) of ESTDAB‐026, probed for HLA‐B and C (HC10), CD63, CD9, CD81, and control ER‐resident protein calnexin (CXN). (b) NTA analysis of particles released by cell line ESTDAB‐026. One comparative graph of at least three recordings is shown, with mean and mode indicated. (c) Flow cytometry of cell surface HLA‐A, B and C as detected by antibody W6/32 compared to control second stage FITC anti‐IgG alone (Negative control).

Immunoblot analysis was performed on cell and EV lysates of ESTDAB‐026 cells after 48 h of growth in serum free conditions (Figure 1A). EVs were enriched for HLA‐I (HLA‐B and C, detected with mAb HC10) and CD9, but were low for CD63 and CD81. The EV preparation was also negative for ER–resident calnexin. NTA of 24 h serum‐free supernatant identified the presence of sub‐200 nm particles with a mean and mode of 196 and 189 nm, respectively (Figure 1B). Cell surface flow cytometry analysis using the monoclonal antibody W6/32 (Figure 1C) confirmed the expression of HLA‐A, B and C molecules on the cell line. Further, we performed transmission electron microscopy (TEM) on ESTDAB‐026 EV isolated by ultracentrifugation which confirmed the presence of typical cup‐shaped vesicles (Figure S1). In addition, we performed flow cytometry on ESTDAB‐026 EV after overnight absorption to CD9‐magnetic beads. Positive staining was observed with antibodies recognising CD9 and HLA‐I, but not the negative control T‐cell receptor protein CD3 (Figure S2). This confirms expression and correct orientation of HLA‐I on ESTDAB‐026 EV. Taken together, this characterisation data confirms the vesicles isolated from this cell lines as typical of a population of EVs based on MISEV guidelines.

### 3.2 ESTDAB‐026 cell surface versus extracellular vesicle HLA‐I immunopeptidome

Our previously established protocol (Kumar et al., 2022) was used to investigate the HLA‐immunopeptidome of both cell and EV lysates. This optimises the speed of cell and EV lysate HLA‐I immunoisolation to conserve lower affinity peptide interactions. To confirm the status of the EV isolated by ultracentrifugation compared to those directly studied by NTA shown in Figure 1, we analysed EV containing cell supernatant pre‐and post‐ultracentrifugation. No significant difference in size or distribution of particles was detected (Figure S3). HLA‐I peptide samples were analysed by mass spectrometry, using a Sciex TripleTOF 5600 instrument, and peptides were identified using Mascot DB software. Eluted peptides from independent replicates of cell or EV lysates were combined, and duplicate peptides removed to obtain a complete list of unique 8 and 15 mer eluted peptides from either ESTDAB‐026 cells or EVs. Details of relevant peptides identified are presented in a subsequent section.

**FIGURE 2:**
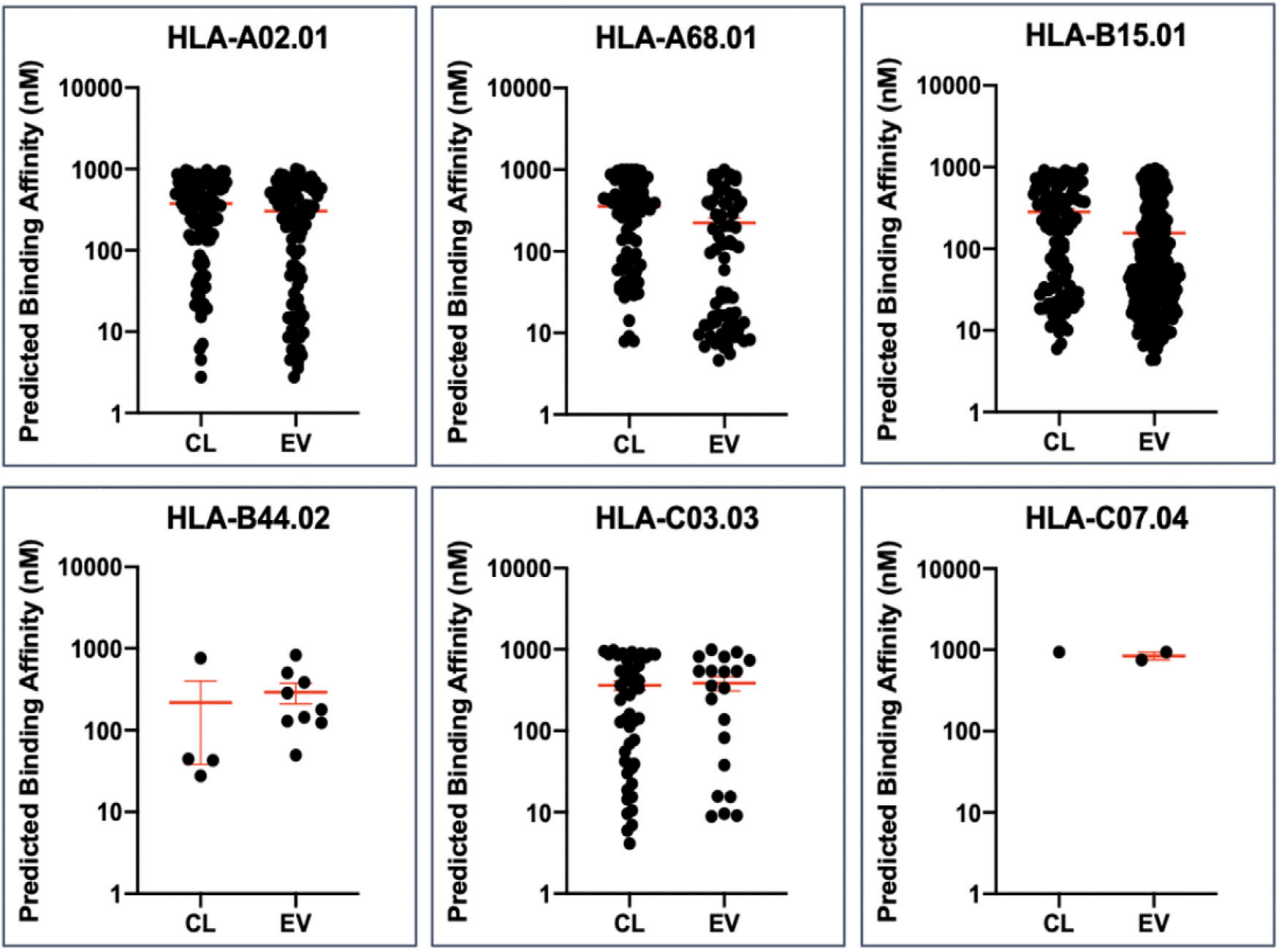
Predicted HLA‐A, ‐B and ‐C binding affinities of Mascot DB peptides from cell (CL) and EV lysates (EV) of ESTDAB‐026. Determined using algorithm netMHCpan 4.0 for HLA genotypes HLA‐A02.01, HLA‐A68.01, HLA‐B15.01, HLA‐B44.02, HLA‐C03.03 and HLA‐ C07.04. Each dot represents a single identified peptide. Data are shown as mean predicted affinity ± S.E (shown in red) for each dataset.

A total of 3590 cell‐derived and 2485 EV‐derived peptides were identified (Table 2). After size and HLA‐I binding affinity prediction algorithm (netMHCpan 4.0) analysis, 315 and 482 peptides were characterised as HLA‐I binding for cells and EV respectively. The predicted binding affinity distribution of these peptides’ affinity to the six cell line specific alleles (HLA‐A02.01, HLA‐A68.01, HLA‐B15.01, HLA‐B44.02, HLA‐C03.03 and HLA‐C07.04) is presented in Figure 2. There is a notable ‘teardrop’ pattern discernible in the data from alleles HLA‐A2.01, ‐A68.01 and ‐B15.01 that indicates a pool of high affinity peptides.

**TABLE 2:** The HLA‐I ligandome of ESTDAB‐026 cells and extracellular vesicles using Mascot DB analysis.

	Total number of eluted peptides	Number of peptides in HLA‐I ligandome (8‐ 15‐mer peptides)	Number of netMHCpan 4.0 binding peptides	Number of known immunogenic peptides identified in the study	Number of peptides derived from known TAA (TAApep)
Cell lysate	3590	2914	315	2	5
EV lysate	2485	2097	482	2	15

Details of total eluted, 8–15 mer peptides, netMHCpan 4.0 predicted binding peptides, previously reported T‐cell epitopes and peptides derived from TAA proteins from the cell surface and EV ligandome.

### 3.3 Identification of T‐cell epitopes and peptides derived from tumour‐associated antigens isolated from the HLA‐I molecules of ESTDAB‐026 cell surface and extracellular vesicles

Peptides identified on the cell surface and the EV immunopeptidomes were searched to establish the presence of tumour antigenic peptides and determine whether any of those identified could be considered immunogenic based on previous reports of T cell activity. The cell and EV immunopeptidome were searched using the Tantigen database. The number of identified T‐cell epitopes from cell and EV ligandomes identified with Mascot DB are presented in Table 3, respectively. A total of two peptides from the cell immunopeptidome were identified to match known T‐cell epitopes. In the EV immunopeptidome, the same two peptides were also detected (Table 3). There is direct potential clinical relevance for these T‐cell epitopes, with peptide ETIPLTAEKL being an epitope within Cyclin‐D1 (CCND1). CCND1 is a classified oncogene, known to be implicated in the promotion of uncontrolled cell proliferation and has been described to be highly expressed in melanoma (González‐Ruiz et al., 2020).

**TABLE 3:** Details of known T‐cell epitopes/immunogenic peptides identified in HLA ligandome of ESTDAB‐026, according to Mascot DB analysis.

Number	Antigen name	Epitope ID	Position	Uniprot entry name	T‐cell epitope	HA‐I allele	Peptides identified in the study
1	Cyclin DI	T000697	115‐124	CCND1_HUMAN	ETIPLTAEKL	HLA‐A*6801	ETIPLTAEKL
2	SRY (sex determining region Y)‐box 10	T000553	332‐340	SOX10_HUMAN	AWISKPPGV	HLA‐A*2	ISKPPGVAL*

T‐cell epitopes/immunogenic peptides detected from the cell surface and EV HLA ligandome. Peptides showing partial match with T‐cell epitopes are marked with an asterisk (*).

The peptides identified on the cell and EV ligandomes of ESTDAB‐026 were then searched for the presence of peptides that could be identified as being derived from known tumour associated antigen (TAA) proteins. These TAA proteins are known to be upregulated in cancer, however, many of the HLA‐I binding peptides of these particular proteins have not been identified previously. Therefore, the peptides identified to be derived from the TAA proteins may or may not be antigenic. Tantigen database was used to identify peptides derived from the full‐length protein sequences of TAA. A total of five peptides were identified to be derived from TAA on the cell surface ligandome (Table S1). In the EV ligandome, a total of 15 peptides were identified to be derived from TAA (Table S2). In summary this data indicates that HLA‐I on EV can display potentially clinically relevant antigenic peptides.

### 3.4 The HLA‐I ligandome of melanoma patient blood derived extracellular vesicles

**FIGURE 3:**
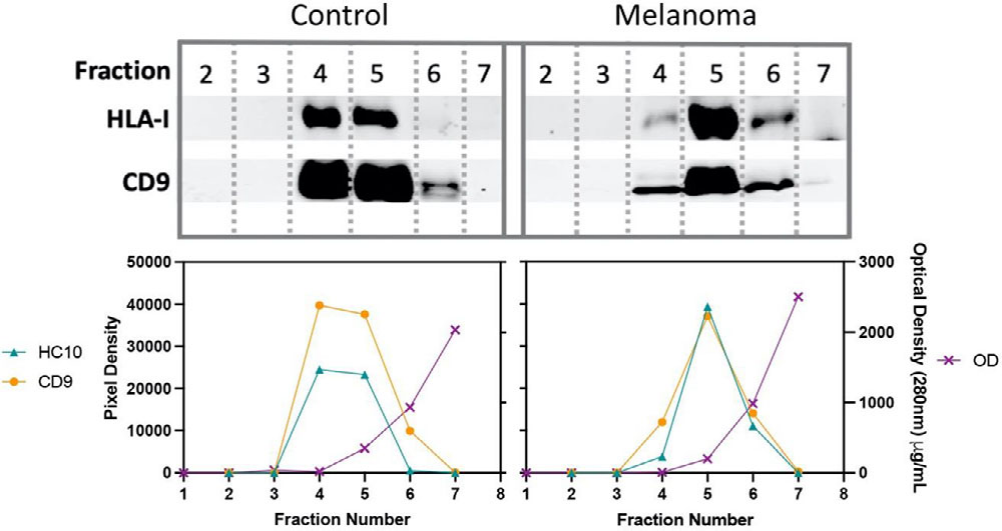
Confirmed presence of extracellular vesicles as a result of size exclusion chromatography (SEC) and immunoblot analysis from melanoma patient plasma samples. Immunoblot analysis of resuspended fractions 2–7 obtained by SEC of 1 representative melanoma plasma sample and 1 representative healthy control plasma sample probed for HLA‐B and C (HLA‐I) and CD9 (for comprehensive presentation of all sample immunoblots, see Figure S5). Lower figure shows graphic presentation of the pixel density, according to the immunoblots in upper figure, of HLA‐I (HC10) and CD9 and the optical density (OD), read at 280 nm, of each sample obtained by spectrophotometry.

To determine if our findings with in vitro cell line derived EVs could also apply to plasma derived EVs, we studied the HLA‐I immunopeptidomes of EVs isolated from both normal control and melanoma patient plasma samples. Platelet‐depleted plasma was processed by size exclusion chromatography (SEC) to generate 10 fractions, in which fractions 4–6 typically contained EVs expressing CD9 and HLA‐I as determined by immunoblotting (Figures 3 and S5). The pixel density obtained from the immunoblots was plotted against the optical density measurements of eluted fractions, indicating that the EV elute towards the front of the main bulk of plasma proteins. Further, we performed transmission electron microscopy (TEM) on healthy control plasma derived EV isolated by SEC to confirm the presence of vesicles (Figure S1). Sizes of particles eluting in the SEC were also analysed by NTA and averaged mean sizes of 178 nm and modes of 138 nm (Figure S4). An assessment for potential lipoproteins co‐eluting with EV in SEC, which is common in this technique, was not performed in this current study. However, lipids and lipoproteins do not carry HLA‐I molecules and are therefore unlikely to impact our immunopeptidome analysis. Duplicate plasma samples were re‐run, fractions 4–7 pooled, detergent lysed and immunoisolated with W6/32 beads, followed by peptide extraction and mass spectrometry as above for the cell line study.

**FIGURE 4:**
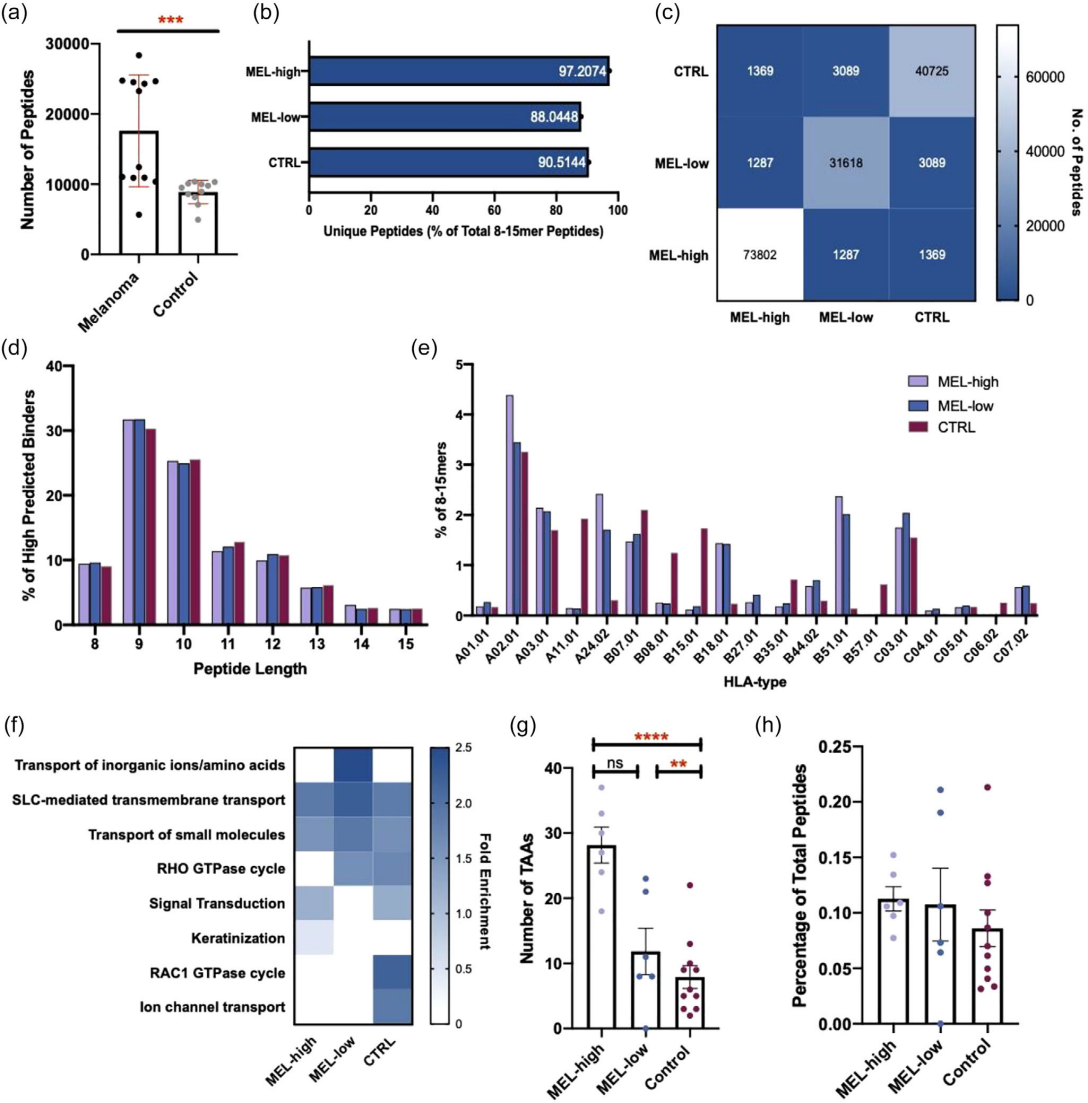
Peptides present on the HLA‐I ligandome of EVs derived from melanoma patient plasma stratify into two different peptidomic profiles. (a) Total peptide yield of melanoma patient samples versus controls. Melanoma patients stratified into two distinct groups: MEL‐high and MEL‐low. (b) Yield of unique peptides in each group. (c) Heat map of peptide overlap between the 2 patient groups and the controls. (d) Distribution of the lengths of peptides deemed to have high predicted binding affinity for HLA‐I. (E) Distribution of peptides with high predicted binding affinity to the 19 most common HLA types. (f) Functional profiles of predicted high HLA‐I binding peptides according to statistical overrepresentation test, carried out by Panther Classification System. (g) Yield of peptides identified to be derived from TAAs, presented as total number achieved by each sample. (h) Yield of peptides identified to be derived from TAAs, presented as a percentage of total peptides yielded by each sample. Data are presented as the mean ± S.E. and unpaired *t*‐tests were performed to assess significance (*p* < 0.05).

The overall number of peptides detected in the HLA‐I immunopeptidome of the melanoma plasma derived EVs was significantly higher than that of the controls (Figure 4A). We also noted that the melanoma plasma samples stratified into two clear distinct groups, with half of the samples displaying a high number of peptides (referred herein as MEL‐high) and the other half resulting in the detection of a lower number of peptides (referred herein as MEL‐low), with the latter similar to that detected in the control samples (Figure 4A). Upon comparison of the unique peptides detected in the MEL‐high, MEL‐low and control samples, MEL‐high samples resulted in a substantially higher percentage of unique peptides (Figure 4B), whilst MEL‐low yielded a higher overlap of peptides with the control samples (Figure 4C). To analyse the potential differences between the two patient groups (MEL‐high vs. MEL‐low) peptide length distribution was assessed, with no significant variation observed between the sample groups (Figure 4D). Analysis of the distribution of peptide binding affinity to the 19 most common UK HLA‐I types showed variations in the number of peptides with high predicted binding affinity (Figure 4E). To investigate the existence of functional variations between the groups, a statistical overrepresentation test was used to identify functional pathways that were significantly altered in each sample group (Figure 4F), with MEL‐high, MEL‐low and the control group displaying varying functional profiles. Several of the pathways identified as being overexpressed are also of clinical significance and relevance to melanoma. For example, the overexpression of keratinization identified in the MEL‐high data impacts on keratinocyte differentiation and their local epidermal microenvironment (Kodet et al., 2015). The HLA‐I immunopeptidomes were then searched for the presence of additional peptides derived from TAA proteins. Significantly more TAAs were detected in MEL‐high group after initial analysis (Figure 4G), however, when adjusted for the total number of peptides yielded, the number of TAAs did not vary significantly between MEL‐high, MEL‐low or the control group (Figure 4H). Full details of the peptides identified to be derived from TAAs are presented in Table S3.

### 3.5 Identification of T‐cell epitopes isolated from the HLA‐I molecules of melanoma patient blood derived extracellular vesicles

Finally, peptides that were identified on the melanoma plasma derived EV immunopeptidomes were searched for tumour antigens and reported T cell activity. A total of 10 peptides from the EV ligandome were identified to match known T‐cell epitopes, according to the Tantigen database search (Table 4).

**TABLE 4:** Details of known T‐cell epitopes/immunogenic peptides identified on HLA ligandome of EVs isolated from melanoma patient plasma samples.

Number	Antigen name	Position	Uniprot entry name	T‐cell epitope	HA‐I allele	Peptides identified in the study	Melanoma patient sample ID
1	Melanoma‐associated antigen 3	149‐160	MAGEA3_HUMAN	VIFSKASSSLQL	Multiple	KASSSLQLVF	MEL‐2
2	Tumour suppressor p53	125‐134	TP53_HUMAN	TYSPALNKMF	HLA‐A*24.02	TYSPALNKMF*	MEL‐2
3	Tyrosinase	386‐406	TYR_HUMAN	FLLHHAFVDSI FEQWLRRHRP	HLA‐DR15	FLLHHAFV*	MEL‐2
4	Milk fat globule‐EGF factor 8	5‐13	MFGE8_HUMAN	RLLAALCGA	HLA‐A*02	MPRPRLLAALCGAL	MEL‐3
5	Calcitonin gene‐related peptide 1	9‐17	CALCA_HUMAN	FLALSILVL	HLA‐A*02.01	SPFLALSILVLL	MEL‐3
6	Lysine Methyltransferase 2A	747‐755	KMT2A_HUMAN	EPRSPSHSM	HLA‐B*07.02	SPIRSEPRSPSHSMR	MEL‐5
7	Squamous cell carcinoma antigen recognized T‐cells 3	302‐310	SART3_HUMAN	LLQAEAPRL	HLA‐A*02	ALLQAEAPRL	MEL‐6
8	Telomerase reverse transcriptase	672‐686	TERT_HUMAN	RPGLLGASVLGLDDI	HLA‐DR7	LLGASVLGL*	MEL‐7
9	Lymphocyte antigen 6 complex, locus K	119‐142	LY6K_HUMAN	KWTEPYCVIA AVKIFPRFFMVAKQ	HLA‐DPB1*0501	CVIAAVKIF*	MEL‐11
10	Non‐muscle alpha‐actinin 4	525‐533	ACTN4_HUMAN	AIDQLHLEY	HLA‐A*01	AIDQLHLEY	MEL‐12

Immunogenic peptides identified by Tantigen database. Peptides showing partial match with T‐cell epitopes are marked with an asterisk (*).

## 4 DISCUSSION

The characterisation of the HLA‐I immunopeptidome landscape of both normal and tumour derived EVs will potentially provide molecular signatures associated with both health and disease, and in the latter case, may also provide a library of potential immunotherapy target antigens. To date, however, there is a significant lack of comprehensive knowledge of EV HLA‐I immunopeptidomes. Whilst a range of diseases may be potentially reported on by studying the EV HLA‐I immunopeptidome, as impacted cells alter their protein expression, it is perhaps in the area of cancer where significant observations may be made. The data presented in this study suggests that the HLA‐I immunopeptidome of cancer EVs can be successfully characterised for EVs derived from melanoma patient blood samples. It is also confirmed that it is possible to identify both T cell epitopes and TAA peptides derived from TAA proteins in EV immunopeptidomes.

The ability to obtain valuable information in the context of cancer from a readily accessible biofluid is an attractive prospect. The results presented in this study highlight the extent of information that the HLA‐I ligandomes of EVs present in patient blood can offer and the fact that these EVs can be obtained relatively non‐invasively, without the need for a more invasive or biopsy, means that they could be considered a repeatable source of information regarding the state of disease over time. The presence of T cell epitopes in a number of the melanoma patient plasma samples investigated further confirms that there are clinically relevant and immunogenic antigens present on the HLA‐I ligandome of plasma EVs obtained from melanoma cancer patients. The detection of these immunogenic peptides on the HLA‐I ligandome of EVs derived from the blood of melanoma patients also further raises the possibility that EVs are utilised by cancer cells as a method of immune evasion as we have previously suggested (Kumar et al., 2022). As such EVs enter the circulation and typically travel away from the cancer cell site of origin, they could potentially activate or alter T cells off‐target, thus reducing direct tumour cell death. There have been very few studies on the ability of HLA‐I peptides on EVs to activate CD8+ T cells, however, one study investigated the ability of EVs to activate CD8+ T cells with common antigenic viral peptides (Admyre et al., 2006). IFNγ secretion was detected, thus indicating cytotoxic T cell activation by EVs and therefore, it is reasonable to predict that cancer derived EVs are also able to directly activate T cells.

Whilst known T cell epitopes and peptides derived from known TAAs were identifiable in the HLA‐I ligandome of blood derived EVs, there is not yet an efficient algorithm or data analysis method to easily identify tumour‐specific antigens (TSAs) or neoantigens. TSAs are known to be derived from various forms of non‐synonymous somatic mutations including single nucleotide variations, frameshift mutations, structural mutations, fusion genes and insertions and deletions (Greenman et al., 2007). These non‐synonymous somatic mutations occur during tumorigenesis due to the occurrence of DNA mutations of the cells that subsequently results in the dysfunctionality of peptide synthesis (Wirth & Kühnel, 2017). As a result, TSAs are typically only expressed by cancer cells. The EVs released from cancer cells carry many of the same peptides as that of the cancer cell surface HLA‐I molecules and therefore, it is very likely that TSA/neoantigens will also be carried by EVs. As many of the peptide assignment analysis techniques require reference databases, it is more difficult to identify these TSAs from mass spectrometry data. Improved methods and algorithms to identify such TSA peptides efficiently is required to fully capitalise on the EV‐based analysis we report here.

To date, no previous reports show overall alterations in the HLA‐I immunopeptidome of EVs in cancer patients. This study indicates that melanoma patients may be stratified into two various immunological profile groups based on the numbers of peptides present on the HLA‐I ligandomes of blood derived EVs. Further study on more samples is required to understand why this is occurring. Whilst cancer patient stratification based on the EV immunopeptidome has not been described before, different EV‐RNA profiles have been ascribed to responders and non‐responders of chemotherapeutic agent dacomitinib in patients diagnosed with recurrent glioblastoma (Yekula et al., 2021). Further exploration with patient samples that are fully characterised regarding the treatment regimens received and associated clinical outcomes will be required for a full understanding of the importance of immunopeptidome stratification in melanoma patients.

T cell epitopes identified in the EV immunopeptidome of our samples are of clinical relevance. The peptide KASSSLQLVF, detected in the EV ligandome of MEL‐2, is a known T cell epitope of Melanoma‐associated Antigen 3 (MAGE‐A3), which is a cancer testis antigen that has been well characterised for its’ role in melanoma as a tumour antigen (Boon & van der Bruggen, 1996; Brasseur et al., 1995). Due to the high immunogenicity of this antigen, there have been a number of clinical trials that utilise anti‐MAGE‐A3 immunotherapy in the treatment of metastatic melanoma (Hersey et al., 2004; Palucka et al., 2003; Schuler‐Thurner et al., 2002). Further, the peptide ALLQAEAPRL, detected in the EV immunopeptidome of sample MEL‐6, is a known T cell epitope of squamous cell carcinoma antigen recognized by T‐cells 3 (SART‐3). SART‐3 is a nuclear protein that is known to have involvement in tumour immunogenicity, particularly in the context of melanoma, and is involved in the regulation of several oncogenic proteins (Whitmill et al., 2016). It has also been shown that high expression of SART‐3 in melanoma patients is associated with a poorer overall survival rate, likely due to its’ substantial role in the regulation of inflammatory cytokine interleukine‐8 (Timani et al., 2018).

At present, a full map of the ‘normal’ EV HLA‐I immunopeptidome and that of cancers has not been fully explored. The data presented in this report alongside our previous study (Kumar et al., 2022), highlights the significance of studying the peptide repertoire found on EVs. Whilst there are still many questions relating to the clinical relevance and disease outcome of these novel findings, we propose that fully exploring the plasma EV HLA‐I immunopeptidome in both health and disease will prove to be a source of highly useful information.

## AUTHOR CONTRIBUTIONS

**Caitlin Boyne**: Data curation; formal analysis; investigation; methodology; writing—original draft; writing—review and editing. **Abigail Coote**: Investigation. **Silvia Synowsky**: Investigation. **Aaron Naden**: Investigation. **Sally Shirran**: Funding acquisition; investigation; writing—review and editing. **Simon Powis**: Conceptualization; funding acquisition; methodology; project administration; supervision; writing—review and editing.

## CONFLICT OF INTEREST STATEMENT

The authors declare no conflicts of interest.

## Supporting information

Supplementary Information

## Data Availability

The mass spectrometry proteomics data have been deposited to the ProteomeXchange Consortium via the PRIDE partner repository with the dataset identifier PXD044822 an https://doi.org/10.6019/PXD044822.
