# Supplementary material for: Characterising the HLA‐I immunopeptidome of plasma‐derived extracellular vesicles in patients with melanoma
Source: J Extracell Biol. 2024 Mar 26;3(3):e146. doi: 10.1002/jex2.146 (PMC11080910; doi:10.1002/jex2.146)
Supplement: Supplementary file 1 — Supplementary Information [file JEX2-3-e146-s001.docx]

**Supplementary Data**

**Characterising the HLA-I Immunopeptidome of plasma-derived extracellular vesicles in patients with melanoma.**

**Caitlin Boyne^1^, Abbie Coote^1^, Silvia Synowsky ^2,3^, Aaron Naden^4^, Sally Shirran ^2,3^ and Simon J. Powis ^1,3^.**

**1. School of Medicine, University of St Andrews, Fife, Scotland, KY16 9TF**

**2. School of Biology, University of St Andrews, Fife, Scotland, KY16 9ST**

**3. Biological Sciences Research Complex, University of St Andrews, Fife, Scotland, KY16 9ST**

**4. School of Chemistry, University of St Andrews, Fife, Scotland, KY16 9ST**

***Transmission electron microscopy of ESTDAB-026 EV and control plasma sample EV isolated by size exclusion column.***

***
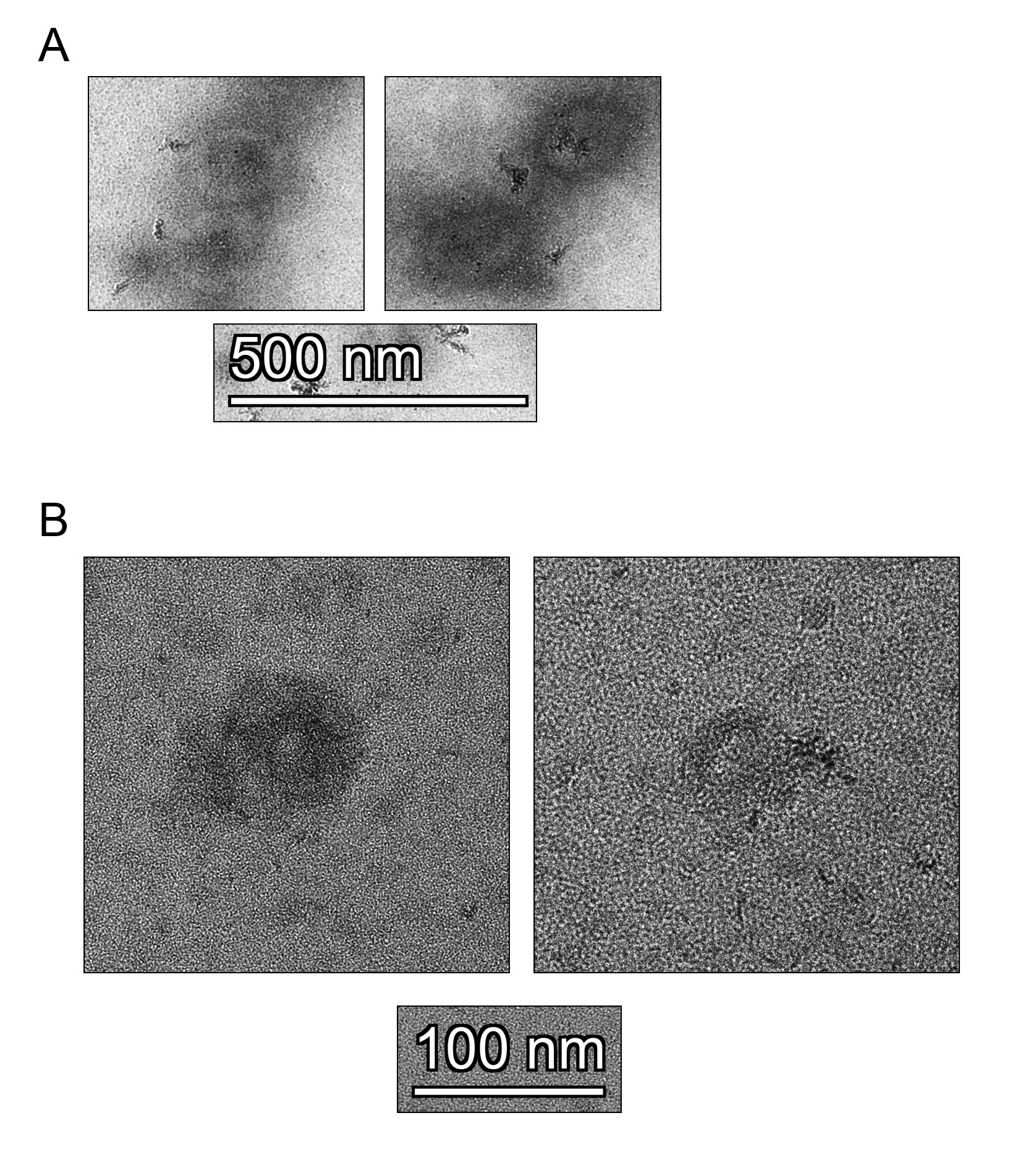
***

***Supplementary Data Figure S1.*** ***Transmission electron microscopy of ESTDAB-026 EV and control plasma sample EV isolated by size exclusion column.*** EV morphology is observed by UA-Zero EM staining in A) ESTDAB-026 EV and B) donor plasma EV isolated by SEC.

***Methods***

In brief, EV isolated by ultracentrifugation for ESTDAB-026 as per main methods section were resuspended in PBS. Plasma EV were isolated by SEC in PBS. EV samples were fixed for 10 mins by mixing with an equal volume of 4% paraformaldehyde. 20 ul samples were placed on parafilm and TEM grids (glow discharged Formvar film on copper grid, Agar Scientific, UK) placed inverted onto the sample for 10 mins. The grids were then placed onto 20 ul drops of UA-Zero EM stain (Agar Scientific, UK) for 10 mins. Grids were then washed by dipping in PBS three times, and then dried on filter paper. Transmission electron microscopy (TEM) measurements were performed on a FEI Titan Themis operated at 200 kV and equipped with a 4k x 4k Ceta CMOS camera.

***Bead based flow cytometry of ESTDAB-026 EV.***

***
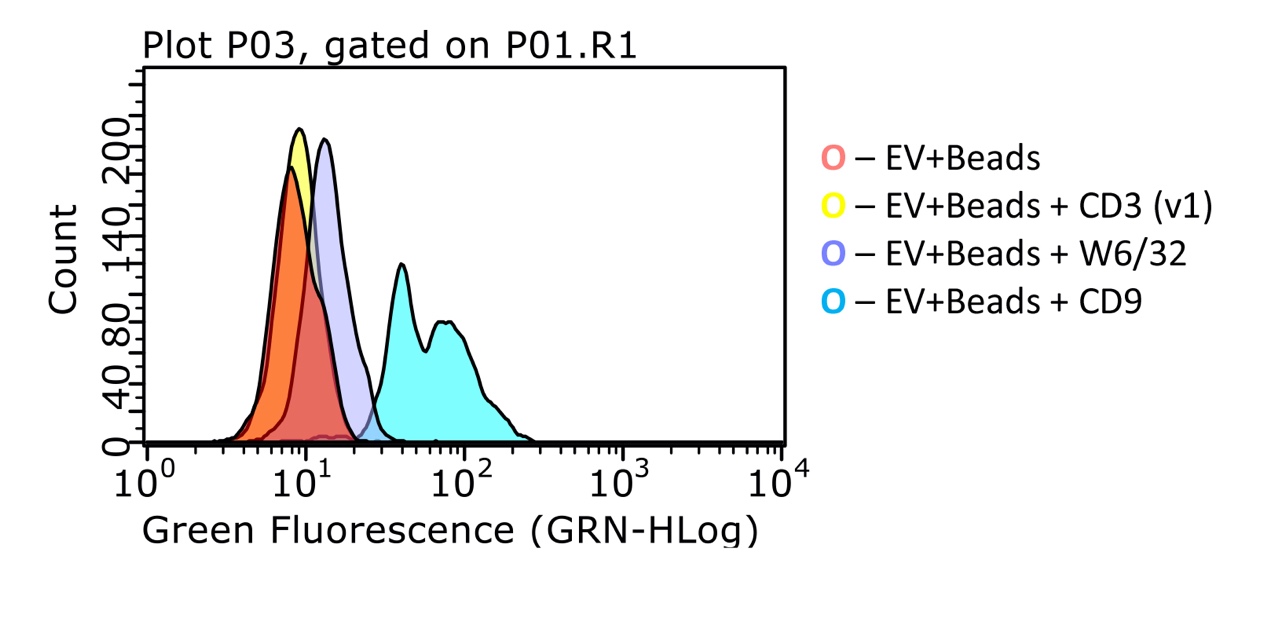
***

***Supplementary Data Figure S2. Bead based flow cytometry of ESTDAB-026 EV.***

EV isolated from the cell culture supernatant of ESTDAB-026 cells were immunoisolated overnight with magnetic beads coated with anti-CD9 antibody (the predominant tetraspanin expressed on these EV, see main text Figure 1). Aliquots of the beads were then labeled with FITC-labelled antibodies to CD9 (pale blue), HLA-I (W6/32, purple line), or an isotype and negative control antibody against the T cell receptor component CD3 (yellow line). EV-coated beads alone are shown in orange. The data confirms the expression and external orientation on the EV of HLA-I and CD9.

***Methods***

ESTDAB-026 cells were cultured for 24-48 hours in RPMI 1640 supplemented with EV depleted 2.5% fetal bovine serum (FBS) (ThermoFisher Scientific, UK). The conditioned media was then collected and centrifuged at 283 x g for 10 mins at 4oC to remove cells and large debris, and then filtered with 0.22 μm Millex-GP syringe filters. 24 mls of supernatant was ultracentrifuged at 124,436 x g for 1 hours in a SW55Ti rotor in a Beckman L100. The EV were resuspended in 0.5 ml PFN ((PBS, 2% FBS, 0.1% Na azide). 0.1 ml of anti-CD9 beads (Invitrogen 10614D) was added and rotated overnight at 4^O^C. The beads were washed twice on a magnetic separator with 1 ml PFN and resuspended in 0.5 ml PFN. 0.1 ml aliquots of beads were incubated with 1 ul of the following antibodies (CD9-FITC - product AbSerotec MCA2655F, W6/32-FITC - product Invitrogen 11-9983-42, CD3-FITC - product Invitrogen HIT3a) for 1 hour at 4^O^C, After washing with PFN a further three times beads were resuspended in 0.1 ml PFN and analysed on a Merck-Millipore Guava 8HT flow cytometer with a 488 nm laser using Guavasoft 2.7 software.

***NTA analysis of ESTDAB-026 EV pre-and post-ultracentrifugation.***


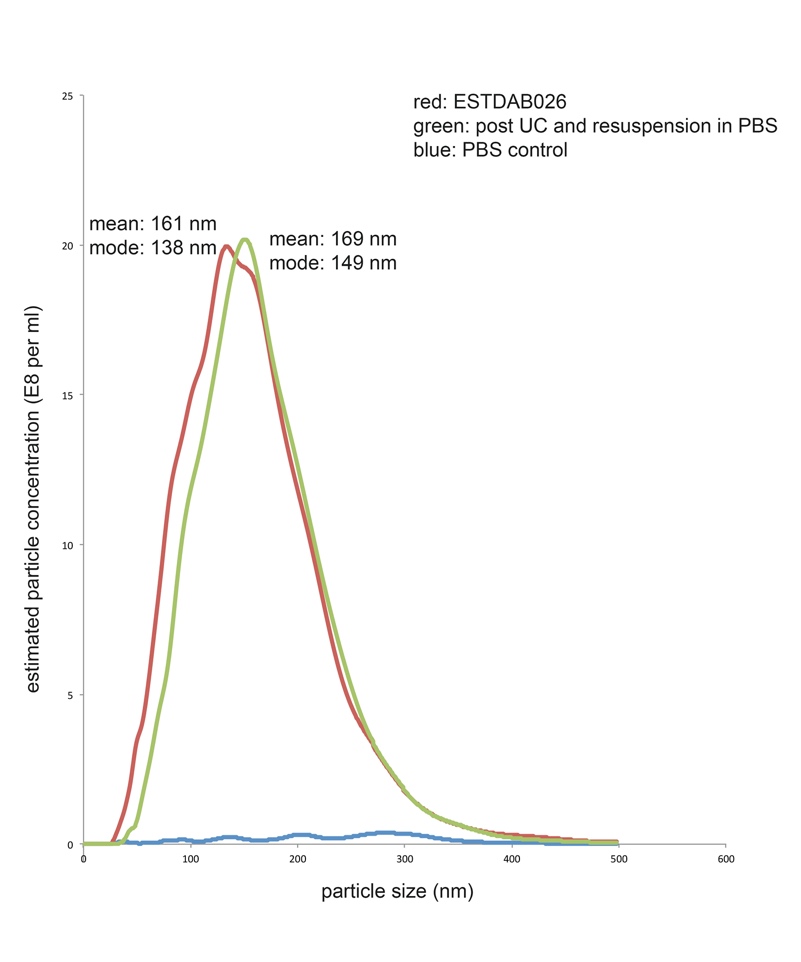


***Supplementary Data Figure S3. NTA analysis of ESTDAB-026 EV pre-and post-ultracentrifugation.*** EV containing supernatant from ESTDAB-026 cells was analysed directly by NTA (red line), or a duplicate sample was ultracentrifuged, resuspended in half the original volume of PBS and analysed (green line). PBS was used as a negative control (blue line). Graphed lines are presented from one set of representative data, whilst the mean and mode for the particles detected are from three independent recordings. No significant alterations in the size or distribution of particles was detected post-ultracentrifugation. The similarity in recovered particles counts, taking into account the resuspension of the ultracentrifuged sample in half its original volume, suggest recovery of around 50% post-ultracentrifugation.

***Methods***

The ESTDAB-026 cell line was cultured in RPMI 1640 supplemented with EV depleted 2.5% fetal bovine serum (FBS) and 50 μg/ml kanamycin (all ThermoFisher Scientific, UK). The FBS was depleted of EVs by ultracentrifugation at 98,380 x g for 4 hours at 4^o^C using a SW32Ti rotor in a Beckman L100 ultracentrifuge, followed by 0.2 μm filtration. ESTDAB-026 EV containing cell supernatants were isolated from cell cultures, and filtered at 0.2 μm. A 4 ml sample was stored for NTA analysis, whilst 4 ml was ultracentrifuged for 1 hour at 124,436 x g in a SW55Ti rotor in a Beckman L100. The EV pellet was resuspended in half the original volume (2 ml PBS) and both samples analysed. NTA was performed using a LM-10 unit (Malvern, UK) equipped with a 638 nm laser. Three videos of 30 seconds length were recorded for each sample with shutter speeds of 30 ms. Data analysis was performed using NTA 2.3 software with detection thresholds of 3. Three videos were recorded from each sample.

***NTA of control plasma sample run on size exclusion column.***


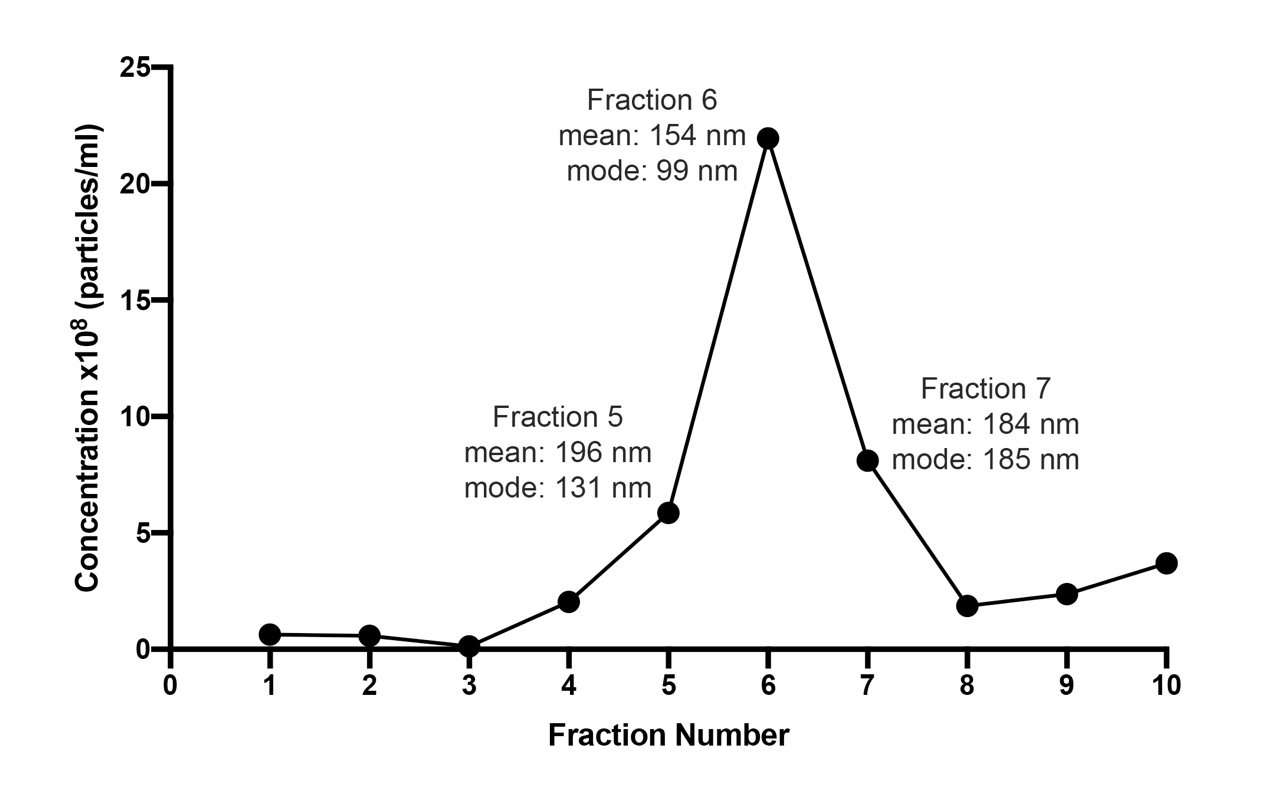


***Supplementary Data Figure S4. NTA of control plasma sample run on size exclusion column.*** A 1 ml sample of plasma from a healthy control donor was run down a 10 ml size exclusion column. Recovered samples were analysed by NTA, and demonstrate typical sizes for EV recovered from plasma. The location of the eluted particles corresponds to the detection of CD9 and HLA-I by immunoblotting as shown in main text Figure 3.

***Methods***

A size exclusion column was prepared as per the main text. Briefly, a 10 ml plastic column (Thermo Fisher Scientific) sealed at the base with fibre glass frits were loaded with Sepharose CL-4B beads (Sigma-Aldrich UK), a second frit placed at the top and the column washed with 20 ml of PBS. 1 ml of the platelet-depleted plasma (centrifuged at 21,130 x g for 5 mins at 4oC ) was then loaded onto the column. Once the sample had passed through the top frit, approximately 12 ml of PBS was added to the top chamber and 10 x 1ml fractions were collected. NTA was performed using a LM-10 unit (Malvern, UK) equipped with a 638 nm laser. Three videos of 30 seconds length were recorded for each sample with shutter speeds of 30 ms. Data analysis was performed using NTA 2.3 software with detection thresholds of 3.

**
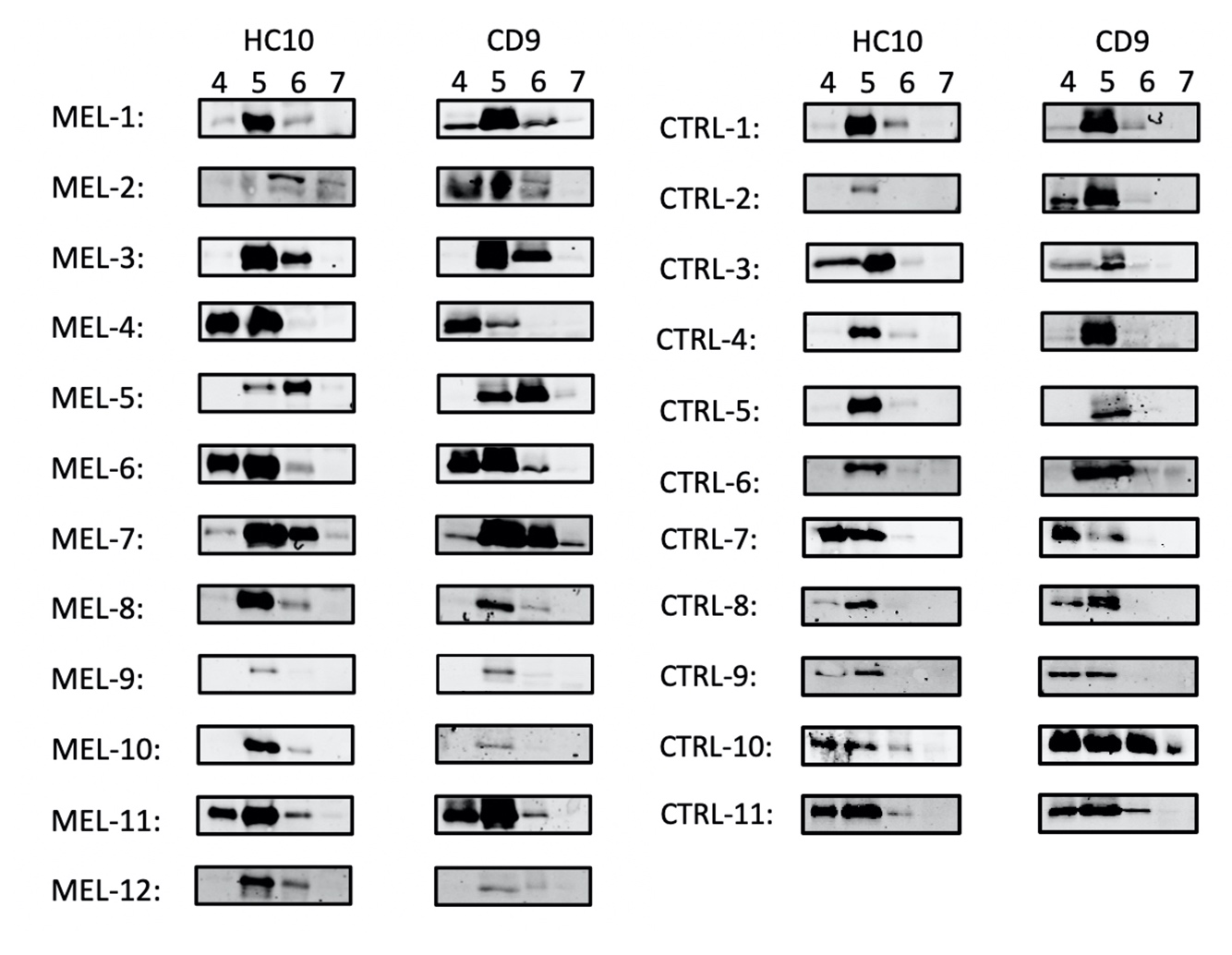
**

***Supplementary Data Figure S5: Confirmed presence of extracellular vesicles as a result of size exclusion chromatography (SEC) and immunoblot analysis.*** Immunoblot analysis of resuspended fractions 4, 5, 6 and 7, obtained by SEC of 12 melanoma plasma samples (MEL-1 – 12) and 11 healthy control plasma samples (CTRL-1 – 11) probed for HLA-B and C (HC10) and CD9.

***Methods***

Methods as per main text section on SEC. 1 ml vials of patient plasma were thawed and centrifuged at 21,130 x g for 5 mins at 4oC to deplete remaining platelets and cell debris. For size exclusion chromatography (SEC) 10 ml plastic columns (Thermo Fisher Scientific) sealed at the base with fibre glass frits were loaded with Sepharose CL-4B beads (Sigma-Aldrich UK), a second frit placed at the top and the column washed with 20 ml of PBS. 1 ml of the platelet-depleted plasma was then loaded onto the column. Once the sample had passed through the top frit, approximately 12 ml of PBS was added to the top chamber and 10 x 1ml fractions were collected. The optical density (280 nm) of each fraction was then assessed by NanoVue Plus Spectrophotometer (Biochrom, Ltd). Fractions of interest were spun at 124,436 x g for 30 mins using a SW55Ti swing-out rotor in a Beckman L100 ultracentrifuge. The resulting EV pellets were resuspended in 30 μl reducing sample buffer. Plasma derived EVs were electrophoresed on 4-20% gradient SDS-PAGE gels (ThermoFisher Scientific, UK) and transferred to nitrocellulose filters (BA85, ThermoFisher Scientific, UK). Membranes were probed with anti-CD9 antibody (ThermoFisher Scientific, UK clone Ts9) at 1:5000 dilution and anti-HLA-B and -C mouse monoclonal antibody HC10 (Stam et al., 1986) at 1:1000 dilution. Immunoblot signals were revealed with 1:10,000 diluted IR Dye800cw anti-mouse (LI-COR, UK) and were then visualized using a LI-COR Odyssey scanner.

***Supplementary Data Table 1: The HLA-I ligandome of ESTDAB-026 cells.*** Details of peptides derived from TAA proteins from the cell surface ligandome including the TAA protein, the Uniprot ID, the sequence of the peptide identified in the study and whether there is a described T cell epitope for the TAA protein, according to Tantigen database.

| **Number** | **Antigen Name** | **Uniprot Entry Name** | **Peptides Identified in the Study** | **T cell epitope defined in Tantigen** |
| --- | --- | --- | --- | --- |
| 1 | Neutral alpha-glucosidase AB | GANAB_HUMAN | AVAAVAARR | Yes |
| 2 | Ryanodine receptor 3 | RYR3_HUMAN | LLAVVVYLYTVV | No |
| 3 | Probable ubiquitin carboxyl-terminal hydrolase FAF-X | USP9X_HUMAN | TVKRLLIKKLPPV | No |

***Supplementary Data Table 2: The HLA-I ligandome of EVs derived from ESTDAB-026.*** Details of peptides derived from TAA proteins from the EV surface ligandome including the TAA protein, the Uniprot ID, the sequence of the peptide identified in the study and whether there is a described T cell epitope for the TAA protein, according to Tantigen database.

| **Number** | **Antigen Name** | **Uniprot Entry Name** | **Peptides Identified in the Study** | **T cell epitope defined in Tantigen** |
| --- | --- | --- | --- | --- |
| 1 | Non-muscle alpha-actinin 4 | ACTN4_HUMAN | KQYERSIVDY | Yes |
| 2 | Annexin A2 | ANXA2_HUMAN | ALSGHLETV | Yes |
| 3 | Annexin A2 | ANXA2_HUMAN | AQRQDIAFAY | Yes |
| 4 | Cyclin D1 | CCND1_HUMAN | VQKEVLPSM | Yes |
| 5 | Neutral alpha-glucosidase AB | GANAB_HUMAN | AVAAVAARR | Yes |
| 6 | eIF-2-alpha kinase activator GCN1 | GCN1_HUMAN | GLVEQYLSAILSL | No |
| 7 | Microtubule-Actin Crosslinking Factor 1 | MACF1_HUMAN | NQKPPSAEY | Yes |
| 8 | myeloid cell leukemia sequence 1, isoform | MCL1_HUMAN | VQRNHETAF | Yes |
| 9 | MIA SH3 Domain ER Export Factor 2 | MIA2_HUMAN | KKLALMLSGL | No |
| 10 | 2'-5'-oligoadenylate synthetase 3 | OAS3_HUMAN | SLAEGLRTV | Yes |
| 11 | Human preferentially expressed antigen of melanoma (PRAME) | PRAME_HUMAN | GQHLHLETF | Yes |
| 12 | SET Domain Containing 2 | SETD2_HUMAN | SSLQSLPPGI | Yes |
| 13 | TPX2, Microtubule Nucleation Factor | TPX2_HUMAN | ITVPKSPAF | Yes |

***Supplementary Data Table 3: The HLA-I ligandome of EVs isolated from plasma of patients diagnosed with melanoma.*** Details of peptides derived from TAA proteins from the EV surface ligandome including the TAA protein, the Uniprot ID, the sequence of the peptide identified in the study and whether there is a described T cell epitope for the TAA protein, according to Tantigen database. EVs were isolated from 12 melanoma patient samples (MEL-1-12) and 11 control human plasma samples (CTRL-1-11).

| **Sample** | **Antigen Name** | **Uniprot Entry Name** | **Peptides Identified in the Study** | **T cell epitope defined in Tantigen** |
| --- | --- | --- | --- | --- |
| MEL-1 | Exportin-1 | XPO1_HUMAN | LLDFSQKL | No |
|  | Met proto-oncogene (hepatocyte growth factor receptor) | MET_HUMAN | SGNSRHISI | Yes |
|  | Suppressor Of Glucose, Autophagy Associated 1 | SOGA1_HUMAN | SLKKRGTRSLG | Yes |
|  | Serine/threonine-protein kinase ATR | ATR_HUMAN | KLKVFREFL | No |
|  | HECT, UBA and WWE domain containing E3 ubiquitin protein ligase 1 | HUWE1_HUMAN | NRRPVMLTLL | No |
|  | Protein kinase with no lysine 2 | WNK2_HUMAN | TSKSKLKAGK | Yes |
|  | Calcium activated chloride channel family member 2 | CLCA2_HUMAN | AGYGSRGRVF | Yes |
|  | Protein kinase with no lysine 2 | WNK2_HUMAN | TYMVEHDFIL | Yes |
|  | Probable E3 ubiquitin-protein ligase HERC1 | HERC1_HUMAN | VMAERLRYAI | No |
|  | Anaplastic lymphoma kinase | ALK_HUMAN | ALKVMEGHGEV | Yes |
|  | BTB (POZ) domain containing 2 | BTBD2_HUMAN | TLYTAKKYAVPAL | No |
|  | Spen Family Transcriptional Repressor | SPEN_HUMAN | RLKGMALVL | Yes |
|  | Human preferentially expressed antigen of melanoma (PRAME) | PRAME_HUMAN | LIGLSNLTHVL | Yes |
|  | Telomerase reverse transcriptase | TERT_HUMAN | RPALLTSRLRF | Yes |
|  | Monocarboxylate transporter 2 | MOT2_HUMAN | RPLGPNQTT | No |
|  | Tensin 3 | TNS3_HUMAN | IVIEPAQLLK | Yes |
|  | Wolf-Hirschhorn syndrome candidate 2 | WHSC2_HUMAN | RTPIPPSRTLLRK | Yes |
|  | Intestinal micin-2 | MUC2_HUMAN | CSILKSSVF | Yes |
|  | Poly(A) polymerase gamma | PAPOLG_HUMAN | VSTSTRTVM | Yes |
|  | Collagen alpha-1(XVIII) chain | COIA1_HUMAN | RAVGLAGTFRAF | No |
|  | Down syndrome cell adhesion molecule-like protein 1 | DSCAML1_HUMAN | LTVKIPAM | No |
| MEL-2 | Chondroitin sulfate proteoglycan 4 precursor | CSPG4_HUMAN | LRKRRVLFV | Yes |
|  | Peroxidasin homolog-fragment | PXDNL_HUMAN | ATITPEELNCER | Yes |
|  | Cleavage and polyadenylation specificity factor subunit 1 | CPSF1_HUMAN | HQSKVITLCL | Yes |
|  | Inositol 1,4,5-triphosphate receptor, type 2 | ITPR2_HUMAN | KMSSFLYIGDI | No |
|  | Melanotransferrin | MF12_HUMAN | FIRPKDCDVLTAV | Yes |
|  | Probable E3 ubiquitin-protein ligase HERC1 | HERC1_HUMAN | VLLARTMVM | No |
|  | Chondroitin sulfate proteoglycan 4 precursor | CSPG4_HUMAN | KMFTLLDVVN | Yes |
|  | RING finger protein 43 precursor | RNF43_HUMAN | ILMTVVGTIFVII | Yes |
|  | Ubiquitin-conjugating enzyme E2 variant 1 | UBE2V1_HUMAN | ILGVVAGAL | Yes |
|  | Telomerase reverse transcriptase | TERT_HUMAN | RPALLTSRLRF | Yes |
|  | RNA Binding Motif Protein 15 | RBM15_HUMAN | FPSNMHLL | Yes |
|  | Matrilin-2 | MATN2_HUMAN | VPRAAIVFT | No |
|  | Promyelocytic leukemia protein | PML1_HUMAN | LQRIRTGSAL | No |
|  | Tensin 3 | TNS3_HUMAN | IVIEPAQLLK | Yes |
|  | Ryanodine receptor 3 | RYR3_HUMAN | TSLIVAALKK | No |
|  | E1A Binding Protein P300 | EP300_HUMAN | KSSLSRGNKK | No |
|  | Adenomatosis polyposis coli | APC_HUMAN | MVYSLLSMLGTHDK | Yes |
|  | Low density lipoprotein-related protein 1 (alpha-2-macroglobulin receptor) | LRP1_HUMAN | VDMGLSTISRAK | No |
|  | Serine/threonine-protein kinase ATR | ATR_HUMAN | KASVCKPFLFLLK | No |
|  | SET Domain Containing 2 | SETD2_HUMAN | KVDSLTLLKC | Yes |
| MEL-3 | Microtubule Associated Protein 1A | MAP1A_HUMAN | AIYQKDEALHV | Yes |
|  | Secernin 1 | SCRN1_HUMAN | KQGLEAMEEI | Yes |
|  | Lymphocyte antigen 6 complex, locus K | LY6K_HUMAN | LALLLVVALPRV | Yes |
|  | Golgin subfamily A member 3 | GOGA3_HUMAN | APLKPPGPL | No |
|  | E3 ubiquitin-protein ligase TRIP12 | TRIPC_HUMAN | GPVKIDPLAL | No |
|  | Probable ubiquitin carboxyl-terminal hydrolase FAF-X | USP9X_HUMAN | VGPRPPKGFV | No |
|  | AT-Rich Interaction Domain 1B | ARID1B_HUMAN | ARNDMPYPY | Yes |
|  | Non-muscle alpha-actinin 4 | ACTN4_HUMAN | FRDGLKLMLL | No |
|  | Ryanodine receptor 3 | RYR3_HUMAN | LLYKLLAAL | No |
|  | SREBF Chaperone | SCAP_HUMAN | LGLATGIVLV | Yes |
|  | Nuclear Receptor Coactivator 1 | NCOA1_HUMAN | ALDGFFFV | Yes |
|  | Signal transducer and activator of transcription 1, 91kDa | STAT1_HUMAN | YIKTELISV | Yes |
|  | HECT, UBA and WWE domain containing E3 ubiquitin protein ligase 1 | HUWE1_HUMAN | LLLAVLNFT | No |
|  | SWI/SNF-related matrix-associated actin-dependent regulator of chromatin subfamily D member 3 | SMRD3_HUMAN | RLTALLLPPDPIV | No |
|  | eIF-2-alpha kinase activator GCN1 | GCN1_HUMAN | VLSSHKVLPL | No |
|  | Matrilin-2 | MATN2_HUMAN | EKMLAGCFLL | No |
|  | Glutamate Metabotropic Receptor 5 | GRM5_HUMAN | KLLKKLTSHL | Yes |
|  | Anaplastic lymphoma kinase | ALK_HUMAN | LRMSWLIRGV | No |
|  | Oculocutaneous albinism II (pink-eye dilution (murine) homolog) | OCA2_HUMAN | AILGAIWLLIL | Yes |
|  | Synaptotagmin-15 | SYT15_HUMAN | YLSRLTVVV | No |
|  | Chondroitin sulfate proteoglycan 4 precursor | CSPG4_HUMAN | YEVQRAPHNGFLSL | Yes |
|  | E3 ubiquitin-protein ligase TRIP12 | TRIPC_HUMAN | IDPLALVQAI | No |
|  | SET Domain Containing 2 | SET2D_HUMAN | RSKTDRDDKYFSY | Yes |
|  | Sarcoma, synovial, X-chromosome-related 1 | SSX1_HUMAN | RIIPKIMPKK | No |
|  | eIF-2-alpha kinase activator GCN1 | GCN1_HUMAN | LLKSPLAAPR | No |
|  | Probable ubiquitin carboxyl-terminal hydrolase FAF-X | USP9X_HUMAN | GAFAKLIVF | No |
|  | HECT, UBA and WWE domain containing E3 ubiquitin protein ligase 1 | HUWE1_HUMAN | RSYVGIATL | No |
|  | MIA SH3 Domain ER Export Factor 2 | MIA2_HUMAN | HSPYGPSPL | No |
|  | NEDD4 Binding Protein 2 | N4BP2_HUMAN | LLKTLTASEM | Yes |
|  | Lysine Methyltransferase 2A | KMT2A_HUMAN | MQGKRKSI | Yes |
|  | Wilms tumor 1 | WT1_HUMAN | MTSQLECMTWN | Yes |
| MEL-4 | Cleavage and polyadenylation specificity factor subunit 1 | CPSF_HUMAN | NLKVRFKKV | Yes |
|  | Chondrosarcoma-associated protein 2 | CSAG2_HUMAN | FPRQPGREKG | No |
|  | Enhancer of zeste homolog 2 (Drosophila) | EZH2_HUMAN | RRIQPVHILTSV | Yes |
|  | eIF-2-alpha kinase activator GCN1 | GCN1_HUMAN | LVFPFLKMV | No |
|  | eIF-2-alpha kinase activator GCN1 | GCN1_HUMAN | VLSSHKVLPL | No |
|  | eIF-2-alpha kinase activator GCN1 | GCN1_HUMAN | KSALLDFYM | No |
|  | eIF-2-alpha kinase activator GCN1 | GCN1_HUMAN | ELLPRVAML | No |
|  | Glypican 3 | GPC3_HUMAN | KLIMTQVSKSL | Yes |
|  | Probable E3 ubiquitin-protein ligase HERC1 | HERC1_HUMAN | TRLLALHVL | No |
|  | HECT, UBA and WWE domain containing E3 ubiquitin protein ligase 1 | HUWE1_HUMAN | LIKAALPL | No |
|  | Insulin Receptor Substrate 2 | IRS2_HUMAN | SLDEYTLMR | Yes |
|  | Kinesin-like protein KIF20B | KI20B_HUMAN | IIETMSSSK | No |
|  | Lymphocyte antigen 6 complex, locus K | LY6K_HUMAN | LALLLVVALPRV | Yes |
|  | Microtubule-Actin Crosslinking Factor 1 | MACF1_HUMAN | SPTRSSSSAS | Yes |
|  | Microtubule-Actin Crosslinking Factor 1 | MACF1_HUMAN | KLLKIGPQLK | Yes |
|  | Mediterranean Fever | MEFV_HUMAN | SEMEMFNVPEL | Yes |
|  | MORC Family CW-Type Zinc Finger 2 | MORC2_HUMAN | IKLSPATPSRK | Yes |
|  | Myosin Light Chain 9 | MYL9_HUMAN | RAKAKTTKK | Yes |
|  | Nuclear Receptor Coactivator 1 | NCOA1_HUMAN | QEELMNTSVY | Yes |
|  | Ryanodine receptor 3 | RYR3_HUMAN | IPRAVASI | No |
|  | SON DNA Binding Protein | SON_HUMAN | SMMSYERSMMSPMA | Yes |
|  | Serine/Arginine Repetitive Matrix 2 | SRRM2_HUMAN | RSRTPLLPRK | Yes |
|  | Topoisomerase (DNA) II beta 180kDa | TOP2B_HUMAN | KADASKKLLK | Yes |
|  | Protein kinase with no lysine 2 | WNK2_HUMAN | KLLNPLVRQLK | Yes |
| MEL-5 | SWI/SNF-related matrix-associated actin-dependent regulator of chromatin subfamily D member 3 | SMRD3_HUMAN | SPAVRPGLAP | No |
|  | Anaplastic lymphoma kinase | ALK_HUMAN | AIGLLWLLPLLL | Yes |
|  | Calreticulin 3 | CALR3_HUMAN | KPEGIHKDV | No |
|  | eIF-2-alpha kinase activator GCN1 | GCN1_HUMAN | RVLAFLSSV | No |
|  | eIF-2-alpha kinase activator GCN1 | GCN1_HUMAN | VLSSHKVLPL | No |
|  | Heterogeneous nuclear ribonucleoprotein L | HNRPL_HUMAN | ASPVVHIRGL | Yes |
|  | Lysine Methyltransferase 2A | KMT2A_HUMAN | APPSPPAVAA | Yes |
|  | Microtubule-Actin Crosslinking Factor 1 | MACF1_HUMAN | KLVSYLSLLRNI | Yes |
|  | Myocyte Enhancer Factor 2D | MEF2D_HUMAN | GSLVTPSLVTSSL | Yes |
|  | Nuclear Receptor Coactivator 1 | NCOA1_HUMAN | QLLPTLEKAAQL | Yes |
|  | Nuclear Receptor Corepressor 2 | NCOR2_HUMAN | KLGGEAAHL | Yes |
|  | p21 (CDKN1A)-activated kinase 2 | PAK2_HUMAN | LLQHPFLKLA | Yes |
|  | Ras Responsive Element Binding Protein 1 | RREB1_HUMAN | AIFKHMPPL | Yes |
|  | U6 snRNA phosphodiesterase | USB1_HUMAN | TRPGDGSHRRGQSPL | No |
|  | Probable ubiquitin carboxyl-terminal hydrolase FAF-X | USP9X_HUMAN | RPRWVVPVLPKG | No |
|  | Wilms tumor 1 | WT1_HUMAN | LNALLPAVPSL | Yes |
|  | Exportin-1 | XPO1_HUMAN | KLNMILVQI | No |
| MEL-6 | Abl interactor 2 | ABI2_HUMAN | WYEGVMNGVTGLF | Yes |
|  | Non-muscle alpha-actinin 4 | ACTN4_HUMAN | FRDGLKLMLL | No |
|  | Non-muscle alpha-actinin 4 | ACTN4_HUMAN | GPSSAGNGAGGGGSM | No |
|  | Bone marrow stromal cell antigen 2 | BST2_HUMAN | VLLGLSALL | Yes |
|  | ATP-dependent Clp protease proteolytic subunit, mitochondrial | CLPP_HUMAN | VGQAASMGSL | No |
|  | Receptor tyrosine-protein kinase erbB-2 | ERBB2_HUMAN | STDVGSCTL | Yes |
|  | Tyrosine-protein kinase receptor FLT3 | FLT3_HUMAN | FLVKCCAY | No |
|  | eIF-2-alpha kinase activator GCN1 | GCN1_HUMAN | VLSSHKVLPL | No |
|  | eIF-2-alpha kinase activator GCN1 | GCN1_HUMAN | KLYRPPPVLDALGRV | No |
|  | eIF-2-alpha kinase activator GCN1 | GCN1_HUMAN | LLEVLGGSHK | No |
|  | eIF-2-alpha kinase activator GCN1 | GCN1_HUMAN | RQAAAIILNIY | No |
|  | eIF-2-alpha kinase activator GCN1 | GCN1_HUMAN | RQAAEVMGRL | No |
|  | Golgin subfamily A member 3 | GOGA3_HUMAN | APLKPPGPL | No |
|  | Glycoprotein NMB | GPNMB_HUMAN | VRSKGLSVF | No |
|  | Probable E3 ubiquitin-protein ligase HERC1 | HERC1_HUMAN | KLLSNHLALA | No |
|  | Probable E3 ubiquitin-protein ligase HERC1 | HERC1_HUMAN | AQRGSLRYLL | No |
|  | Heparanase | HPSE_HUMAN | KPALPPPLMLLLL | Yes |
|  | Inositol 1,4,5-triphosphate receptor, type 2 | ITPR2_HUMAN | KLLEKHITAK | No |
|  | Lysine Methyltransferase 2A | KMT2A_HUMAN | KIIPAPKPK | Yes |
|  | LIM Domain Containing Preferred Translocation Partner In Lipoma | LPP_HUMAN | IPFTVDAGGL | Yes |
|  | Low density lipoprotein-related protein 1 (alpha-2-macroglobulin receptor) | LRP1_HUMAN | LLLLLPLLSALV | No |
|  | Microtubule-Actin Crosslinking Factor 1 | MACF1_HUMAN | ALNEEIVNRKK | Yes |
|  | Microtubule Associated Protein 1A | MAP1_HUMAN | KMGVGRLDMYVLNPV | Yes |
|  | Met proto-oncogene (hepatocyte growth factor receptor) | MET_HUMAN | QPEYCPDPLY | Yes |
|  | MORC Family CW-Type Zinc Finger 2 | MORC2_HUMAN | IKLSPATPSRK | Yes |
|  | tRNA-splicing ligase RtcB homolog | RTCB_HUMAN | QVVDEIFNEYAAK | No |
|  | Splicing factor 3B subunit 1 | SF3B1_HUMAN | ALMDRDLVHR | No |
|  | SON DNA Binding Protein | SON_HUMAN | MMSPMAERSMMSA | Yes |
|  | Sarcoma, synovial, X-chromosome-related 1 | SSX1_HUMAN | HRIIPKIMPKK | No |
|  | Protein SZT2 | SZT2_HUMAN | GLAPALPLS | No |
|  | Topoisomerase (DNA) II alpha 170kDa | TOP2A_HUMAN | AEMSSYHHGEMSLM | Yes |
|  | Transient receptor potential cation channel, subfamily M, member 8 | TRPM8_HUMAN | FTLRLIHIFTV | Yes |
|  | Ubiquitin carboxyl-terminal hydrolase 28 | UBP28_HUMAN | FMQELQYLFALMM | No |
|  | U6 snRNA phosphodiesterase | USB1_HUMAN | TYVPRLVRMKV | No |
|  | Probable ubiquitin carboxyl-terminal hydrolase FAF-X | USP9X_HUMAN | ALSMIIKSL | No |
|  | Exportin-1 | XPO1_HUMAN | LPMLFKVRLLMV | No |
| MEL-7 | Septin 2 | SEPT2_HUMAN | LLKASIPF | Yes |
|  | Neuroblast differentiation-associated protein AHNAK | AHNK_HUMAN | KANVDISAPK | No |
|  | Alkylglycerol monooxygenase | ALKMO_HUMAN | LLKIYTVVQFA | No |
|  | Annexin A2 | ANXA 2 _HUMAN | KLMVALAKGRRA | Yes |
|  | Serine/threonine-protein kinase ATR | ATR_HUMAN | LELASMIPALR | No |
|  | B-Raf proto-oncogene serine/threonine-protein kinase | BRAF_HUMAN | LSKVRSNCPK | No |
|  | Centrosomal Protein 95 | CEP95_HUMAN | MLSKKALAS | No |
|  | Ephrin type-A receptor 2 precursor | EPHA2_HUMAN | LAAAAAAQGK | Yes |
|  | Formin-like 1 | FMNL1_HUMAN | RAKNLAITLRK | Yes |
|  | Forkhead box protein O1A | FOXO1_HUMAN | GPLSQHPPV | No |
|  | Forkhead box protein O1A | FOXO1_HUMAN | LPHTVSTMPH | No |
|  | Neutral alpha-glucosidase AB | GANAB_HUMAN | LLEDRSLLLSV | Yes |
|  | eIF-2-alpha kinase activator GCN1 | GCN1_HUMAN | VLSSHKVLPL | No |
|  | Glypican 3 | GPC3_HUMAN | LQSASMELKF | Yes |
|  | Glycoprotein NMB | GPNMB_HUMAN | VRSKGLSVF | No |
|  | Lysine Methyltransferase 2A | KMT2A_HUMAN | KVAPRPSIPVKQK | Yes |
|  | Lymphocyte antigen 6 complex, locus K | LY6K_HUMAN | LLLVVALPRV | No |
|  | Microtubule-Actin Crosslinking Factor 1 | MACF1_HUMAN | KLLKIGPQLK | Yes |
|  | Ninein | NIN_HUMAN | YKEHLNSM | No |
|  | Proliferation-associated 2G4, 38kDa | PA2G4_HUMAN | LYEKEGEFVAQFKF | No |
|  | RNA Binding Motif Protein 15 | RBM15_HUMAN | YLKQKQAAGVISL | No |
|  | Sushi repeat-containing protein SRPX | SRPX_HUMAN | LVMPVALF | No |
|  | Telomerase reverse transcriptase | TERT_HUMAN | RPALLTSRLRF | Yes |
|  | Topoisomerase (DNA) II beta 180kDa | TOP2B_HUMAN | IIKIVGLQY | Yes |
|  | E3 ubiquitin-protein ligase TRIP12 | TRIPC_HUMAN | EIMREKLLIA | No |
|  | Probable ubiquitin carboxyl-terminal hydrolase FAF-X | USP9X_HUMAN | KALGHPAMLSKVL | No |
| MEL-8 | Abelson murine leukemia viral (v-abl) oncogene homolog 1 | BCR-ABL_HUMAN | PAVSPLLPRK | No |
|  | Annexin A2 | ANXA2_HUMAN | HSTPPSAYGSVK | Yes |
|  | B-Raf proto-oncogene serine/threonine-protein kinase | BRAF_HUMAN | ILLFMGYSTK | No |
|  | Calreticulin 3 | CALR3_HUMAN | GYIKVFPADI | Yes |
|  | Calcium activated chloride channel family member 2 | CLCA2_HUMAN | LTGGLKFFV | Yes |
|  | Cleavage and polyadenylation specificity factor subunit 1 | CPSF1_HUMAN | TKNKFKVL | Yes |
|  | Cleavage and polyadenylation specificity factor subunit 1 | CPSF1_HUMAN | VQVSPLGIRL | Yes |
|  | Discoidin domain receptor family, member 1 | DDR1_HUMAN | YTAPVGQTM | No |
|  | E1A Binding Protein P300 | EP300_HUMAN | GLGLINSMV | No |
|  | ets variant 5 | ETV5_HUMAN | KLIEPEEVA | Yes |
|  | Neutral alpha-glucosidase AB | GANAB_HUMAN | SKRRKLVAI | Yes |
|  | eIF-2-alpha kinase activator GCN1 | GCN1_HUMAN | VLSSHKVLPL | No |
|  | eIF-2-alpha kinase activator GCN1 | GCN1_HUMAN | MGSLAKHLDK | No |
|  | Probable E3 ubiquitin-protein ligase HERC1 | HERC1_HUMAN | TRLLALHVL | No |
|  | Probable E3 ubiquitin-protein ligase HERC1 | HERC1_HUMAN | TLVTGLQGK | No |
|  | Insulin Receptor Substrate 2 | IRS2_HUMAN | SGVKRLSL | Yes |
|  | Microtubule-Actin Crosslinking Factor 1 | MACF1_HUMAN | KSLSQPTPPPMPI | Yes |
|  | Microtubule-Actin Crosslinking Factor 1 | MACF1_HUMAN | KVKEALAGLLVTY | Yes |
|  | Met proto-oncogene (hepatocyte growth factor receptor) | MET_HUMAN | VLAPGILVL | Yes |
|  | MIA SH3 Domain ER Export Factor 2 | MIA2_HUMAN | AMFGASRDY | No |
|  | NEDD4 Binding Protein 2 | N4BP2_HUMAN | ILKATTPK | Yes |
|  | Peptidyl-prolyl cis-trans isomerase B | PPIB_HUMAN | AAALIAGSVF | Yes |
|  | Ryanodine receptor 3 | RYR3_HUMAN | LLYKLLAAL | No |
|  | Secernin 1 | SCRN1_HUMAN | KQEESITVQTM | Yes |
|  | Splicing factor 3B subunit 1 | SF3B1_HUMAN | AILPHLRSLVEI | No |
|  | Salt Inducible Kinase 1 | SIK1_HUMAN | DEQALGIM | Yes |
|  | SKI Proto-Oncogene | SKI_HUMAN | SPAVSASEKEL | Yes |
|  | SON DNA Binding Protein | SON_HUMAN | MLNRAQERIDAWAQL | Yes |
|  | Probable ubiquitin carboxyl-terminal hydrolase FAF-X | USP9X_HUMAN | LLIKKLPPVLA | No |
|  | Probable ubiquitin carboxyl-terminal hydrolase FAF-X | USP9X_HUMAN | GAFAKLIVF | No |
| MEL-9 | Neuroblast differentiation-associated protein AHNAK | AHNK_HUMAN | KLKFGTFGGL | No |
|  | Anaplastic lymphoma kinase | ALK_HUMAN | ALKVMEGHGEV | Yes |
|  | DNA damage-regulated autophagy modulator protein 1 | DRAM1_HUMAN | VVHDGGALLAFV | No |
|  | Probable E3 ubiquitin-protein ligase HERC1 | HERC1_HUMAN | VLLARTMVM | No |
|  | Histone-lysine N-methyltransferase 2D | KMT2D_HUMAN | IPKGEELTY | No |
|  | MORC Family CW-Type Zinc Finger 2 | MORC2_HUMAN | KQKVPLGTF | No |
|  | SON DNA Binding Protein | SON_HUMAN | LAPRPLMLAS | Yes |
|  | Transient receptor potential cation channel, subfamily M, member 8 | TRPM8_HUMAN | KTPNLVISV | Yes |
| MEL-11 | Microtubule-Actin Crosslinking Factor 1 | MACF1_HUMAN | MEQEALVKTL | Yes |
|  | MIA SH3 Domain ER Export Factor 2 | MIA2_HUMAN | KLKVMTELY | No |
|  | Serine/Arginine Repetitive Matrix 2 | SRRM2_HUMAN | TPRTARGKRSLT | Yes |
|  | Serine And Arginine Rich Splicing Factor 8 | SRSF8_HUMAN | YGGSHYSSSGY | Yes |
|  | Synaptonema complex protein 1 | SYCP1_HUMAN | KSEENCNNL | Yes |
|  | Telomerase reverse transcriptase | TERT_HUMAN | RPALLTSRLRF | Yes |
|  | WD repeat domain 46 | WDR46_HUMAN | SSTASLVKRK | No |
| MEL-12 | Caspase 5 | CASP5_HUMAN | KSSDSTFLVLM | No |
|  | Cyclin D1 | CCND1_HUMAN | APSVSYFKCVQK | Yes |
|  | Cell division cycle 27 | CDC27_HUMAN | HLALMNFSW | Yes |
|  | Calcium activated chloride channel family member 2 | CLCA2_HUMAN | KLLVSYLPTT | Yes |
|  | Ephrin type-A receptor 3 | EPHA3_HUMAN | RGIASGMKY | Yes |
|  | Heme oxygenase (decycling) 1 | HMOX1_HUMAN | APGLRQRAS | Yes |
|  | MIA SH3 Domain ER Export Factor 2 | MIA2_HUMAN | GMRPDSNLYG | No |
|  | Ras Responsive Element Binding Protein 1 | RREB1_HUMAN | MMSAVMSVGK | Yes |
|  | SREBF Chaperone | SCAP_HUMAN | VVWIGILVY | Yes |
|  | Telomerase reverse transcriptase | TERT_HUMAN | RPALLTSRLRF | Yes |
| CTRL-1 | E1A Binding Protein P300 | EP300_HUMAN | KSSLSRGNKK | No |
|  | Glutamate Metabotropic Receptor 5 | GRM5_HUMAN | ARILAGSKKK | Yes |
|  | Probable ubiquitin carboxyl-terminal hydrolase FAF-X | USP9X_HUMAN | GAFAKLIVF | No |
|  | ADAM metallopeptidase domain 17 (tumor necrosis factor, alpha, converting enzyme) | ADAM17_HUMAN | NIVGSVLVF | No |
|  | Down syndrome cell adhesion molecule-like protein 1 | DSCAML1_HUMAN | LTVKIPAM | No |
|  | Lysine Methyltransferase 2A | KMT2A_HUMAN | GLKKRPISRL | Yes |
|  | Microtubule-Actin Crosslinking Factor 1 | MACF1_HUMAN | SRLRMPPLI | Yes |
|  | Prostatic acid phosphatase precursor | ACPP_HUMAN | ELKFVTLVF | Yes |
|  | Proton-coupled folate transporter | SLC46A1_HUMAN | SLGLLLQALVSVF | No |
|  | Solute carrier family 35 member A4 | SLC35A4_HUMAN | MSAVMKHGSSITRLF | No |
|  | Lymphocyte antigen 6 complex, locus K | LY6K_HUMAN | LLLVVALPRV | Yes |
|  | Transient receptor potential cation channel, subfamily M, member 8 | TRPM8_HUMAN | ELVLYSLVFV | Yes |
|  | TBC1 domain family member 9B | TBC1D9B_HUMAN | SFLLGKEVSLVV | No |
|  | Melanotransferrin | MF12_HUMAN | FIRPKDCDVLTAV | Yes |
|  | Serine/threonine-protein kinase ATR | ATR_HUMAN | VLLEYGSPKIKSL | No |
|  | SET Domain Containing 2 | SETD2_HUMAN | SLPPGIKVDSLTLL | Yes |
|  | Protein SZT2 | SZT2_HUMAN | RPAAERHLLL | No |
|  | Telomerase reverse transcriptase | TERT_HUMAN | RPALLTSRLRF | No |
|  | Cyclin B1 | CCNB1_HUMAN | LPKPLEKVPM | Yes |
|  | UBX domain containing 5 | UBXD5_HUMAN | VPKAALLL | No |
|  | Nuclear Receptor Corepressor 2 | NCOR2_HUMAN | TPELPLAPRPL | No |
|  | Leucine-rich repeat-containing protein 41 | LRRC41_HUMAN | SPLPILELTRAIV | No |
| CTRL-2 | Cell division protein kinase 4 | CDK4_HUMAN | EIKVTLVF | Yes |
|  | Golgin subfamily A member 3 | GOGA3_HUMAN | APLKPPGPL | No |
|  | Probable E3 ubiquitin-protein ligase HERC1 | HERC1_HUMAN | VLLARTMVM | No |
|  | Peptidyl-prolyl cis-trans isomerase B | PPIB_HUMAN | WVSMANAGK | Yes |
|  | SON DNA Binding Protein | SON_HUMAN | APRSYRIAP | Yes |
|  | Storkhead-box protein 1 | STOX1_HUMAN | LPATQPIPRI | No |
|  | Synaptotagmin-15 | SYT15_HUMAN | KTSAVLGSI | No |
|  | Telomerase reverse transcriptase | TERT_HUMAN | RPALLTSRLRF | Yes |
|  | Probable ubiquitin carboxyl-terminal hydrolase FAF-X | USP9X_HUMAN | KLSVPATFMLVSL | No |
| CTRL-3 | Anaplastic lymphoma kinase | ALK_HUMAN | ALKVMEGHGEV | Yes |
|  | Cleavage and polyadenylation specificity facotr subunit 1 | CPSF1_HUMAN | KTLSLHYF | Yes |
|  | Tyrosine-protein kinase receptor FLT3 | FLT3_HUMAN | KLIQNGFK | No |
|  | Fucosyltransferase 1 | FUT1_HUMAN | WMSEEYADLRDPF | No |
|  | Glycoprotein NMB | GPNMB_HUMAN | RMANSALISVG | No |
|  | Glycoprotein NMB | GPNMB_HUMAN | VVRSKGLSV | No |
|  | Low density lipoprotein-related protein 1 (alpha-2-macroglobulin receptor) | LRP1_HUMAN | ISVDYQDGK | No |
|  | Microtubule-Actin Crosslinking Factor 1 | MACF1_HUMAN | LLHFQNAVEI | Yes |
|  | SON DNA Binding Protein | SON_HUMAN | IAKANAAAM | Yes |
|  | SON DNA Binding Protein | SON_HUMAN | GMGAVLMRK | Yes |
|  | SRY (sex-determining region Y)-box 10 | SOX10_HUMAN | IREAVSQVL | Yes |
|  | Supervillin | SVIL_HUMAN | DETFAKFY | Yes |
|  | Protein kinase with no lysine 2 | WNK2_HUMAN | ILLGHPAPY | No |
| CTRL-4 | Serine/threonine-protein kinase ATR | ATR_HUMAN | FVVLMEIIAKVFL | No |
|  | Inositol 1,4,5-triphosphate receptor, type 2 | ITPR2_HUMAN | NLFLTPGLLEA | No |
|  | MORC Family CW-Type Zinc Finger 2 | MORC2_HUMAN | KSGSMRIGK | Yes |
| CTRL-5 | Lymphocyte cell-specific protein-tyrosine kinase | LCK_HUMAN | IVPLDGKGTLL | No |
|  | MORC Family CW-Type Zinc Finger 2 | MORC2_HUMAN | KSGSMRIGK | No |
|  | SON DNA Binding Protein | SON_HUMAN | AERSMMSAYER | No |
| CTRL-6 | Cyclin-dependent kinase 12 | CDK12_HUMAN | TLPSKPVKK | No |
|  | Nuclear Receptor Corepressor 2 | NCOR2_HUMAN | IRGSITQGI | Yes |
|  | Ras Responsive Element Binding Protein 1 | RREB1_HUMAN | AIFKHMPPLKPK | Yes |
|  | SREBF Chaperone | SCAP_HUMAN | APMPVPSGMLP | Yes |
|  | Protein SZT2 | SZT2_HUMAN | AQLLSILTEVR | No |
| CTRL-7 | Discoidin domain receptor family, member 1 | DDR1_HUMAN | ALLLSNPAY | No |
|  | Epidermal growth factor receptor | EGFR_HUMAN | ATCKDTCPPLMLY | Yes |
|  | Tyrosine-protein kinase receptor FLT3 | FLT3_HUMAN | KLIQNGFK | No |
|  | Glutamate Metabotropic Receptor 5 | GRM5_HUMAN | ATSMDLSDK | Yes |
|  | Microtubule-Actin Crosslinking Factor 1 | MACF1_HUMAN | DEKLLHNVLM | Yes |
|  | Ras Responsive Element Binding Protein 1 | RREB1_HUMAN | HAATRLSL | Yes |
|  | SWI/SNF-related matrix-associated actin-dependent regulator of chromatin subfamily D member 3 | SMRD3_HUMAN | MPSGARMPHQGAPM | No |
|  | SON DNA Binding Protein | SON_HUMAN | VTGGMGAVLMRK | No |
|  | SON DNA Binding Protein | SON_HUMAN | SSYTADRSM | No |
|  | Palmitoyltransferase ZDHHC7 | ZDHC7_HUMAN | ALILCGFQF | No |
| CTRL-8 | Neuroblast differentiation-associated protein AHNAK | AHNK_HUMAN | RVDIETPNL | No |
|  | Receptor tyrosine-protein kinase erbB-2 | ERBB2_HUMAN | LLVVVLGVVF | Yes |
|  | Fibroblast growth factor 5 precursor | FGF5_HUMAN | GIVGIRGVF | No |
|  | Fucosyltransferase 1 | FUT1_HUMAN | GINADLSPL | No |
|  | Histocompatibility (minor) HA-1 | HMHA1_HUMAN | TPGNLGIVF | No |
|  | Hydroxysteroid dehydrogenase like 1 | HSDL1_HUMAN | VICDFYSLIR | No |
|  | Microtubule-Actin Crosslinking Factor 1 | MACF1_HUMAN | QYKLVSDTI | Yes |
|  | Topoisomerase (DNA) II alpha 170kDa | TOP2A_HUMAN | QVLEPMLNGTEK | No |
|  | X-box-binding protein 1 | XBP1_HUMAN | SPWILAVL | No |
| CTRL-9 | BCR-ABL fusion protein | BCR-ABL_HUMAN | MIYLQTLLAKEKK | Yes |
|  | Cyclin-dependent kinase 12 | CDK12_HUMAN | NPAVTAALL | No |
|  | Adenocarcinoma antigen recognized by T lymphocytes 4 | NOB1_HUMAN | AMQNVLLQMGL | No |
|  | Ryanodine receptor 3 | RYR3_HUMAN | QHQFGMDLLL | No |
|  | TPX2, Microtubule Nucleation Factor | TPX2_HUMAN | VIIDEILPSKK | No |
| CTRL-10 | Neuroblast differentiation-associated protein AHNAK | AHNK_HUMAN | KMKGDVVVSLPK | No |
|  | Serine/threonine-protein kinase ATR | ATR_HUMAN | FASILGQLVCTL | No |
|  | Chondroitin sulfate proteoglycan 4 precursor | CSPG4_HUMAN | LLSDSIPHT | No |
|  | Forkhead box protein O1A | FOXO1_HUMAN | FMSNLSLL | No |
|  | Matrix metallopeptidase 14 (membrane-inserted) | MMP14_HUMAN | LLLLLVLAVGLAV | No |
|  | Serine-threonine kinase receptor-associated protein | STRAP_HUMAN | LMTLAHKHIVK | No |
| CTRL-11 | Regulatory Associated Protein Of MTOR Complex 1 | RPTOR_HUMAN | RFLRNSRVRR | No |
|  | Heat Shock Protein Family B (Small) Member 1 | HSPB1_HUMAN | QLGGPEAAK | No |

***List of Reagents***

RPMI 1640 medium (ThermoFisher Scientific, UK, product code 21875-059).

Fetal Bovine Serum (ThermoFisher Scientific, UK, product code A5256701)

Trypsin (0.025%)-EDTA (0.01%) (ThermoFisher Scientific, UK, product code 25200-056)

Kanamycin (ThermoFisher Scientific, UK product code 15160-047).

0.22 μm Millex-GP syringe filters (Merck, UK product code SLGP033RS).

Sodium (Na) azide (Sigma Aldrich UK, product code S8032)

Bradford assay (ThermoFisher Scientific, UK), product code 23228).

4-20% gradient SDS- PAGE gels (ThermoFisher Scientific, UK product code XV04205PK20)

Anti-CD9 beads (ThermoFisher Scientific, UK product code 10614D)

Anti-CD9-FITC (AbSerotec, product code MCA2655F)

W6/32-FITC (ThermoFisher Scientific, UK product code 11-9983-42)

Anti-CD3-FITC clone HIT3a (ThermoFisher Scientific, UK product code 14-0039-80)

Anti-CD9, clone Ts9 (ThermoFisher Scientific, UK product code 10626D)

Anti-CD63, clone Ts63 (ThermoFisher Scientific, UK product code 10628D)

Anti-CD81, clone M38 (ThermoFisher Scientific, UK product code 10630D)

Anti-human calnexin (Abcam UK, product code ab213243)

IR Dye800cw anti-mouse IgG (LI-COR, UK, product code 926-32210)

IR Dye800cw anti-rabbit IgG (LI-COR, UK, product code 926-32211)

FITC-anti-mouse IgG (Sigma-Aldrich UK, product code F2012)

10 ml plastic columns (Sigma-Aldrich UK. Supelco product code 54806)

Sepharose CL-4B beads (Sigma-Aldrich UK, product code CL4B200 and Cytiva product code 17012001)

Pierce mini-protease inhibitor tablets (ThermoFisher Scientific, UK, product code A32955).

Protein G-Sepharose beads (ThermoFisher Scientific, UK, product code 20399).

BS3 crosslinker (ThermoFisher Scientific, UK, product code 21580).

Trifluoroacetic acid (ThermoFisher Scientific, UK, product code 044630).

Acetonitrile (ThermoFisher Scientific, UK, product code 047138.M1).

Pierce C18 100 μl tips (ThermoFisher Scientific, UK, product code 87784)
